# Calcineurin-mediated IL-2 production by CD11c^high^MHCII^+^ myeloid cells is crucial for intestinal immune homeostasis

**DOI:** 10.1038/s41467-018-03495-3

**Published:** 2018-03-16

**Authors:** Andrea Mencarelli, Hanif Javanmard Khameneh, Jan Fric, Maurizio Vacca, Sary El Daker, Baptiste Janela, Jing Ping Tang, Sabrina Nabti, Akhila Balachander, Tong Seng Lim, Florent Ginhoux, Paola Ricciardi-Castagnoli, Alessandra Mortellaro

**Affiliations:** 1 Singapore Immunology Network (SIgN), Agency for Science, Technology and Research (A*STAR), Singapore, 138648 Singapore; 2 Institut Unité de Biologie des Populations Lymphocytaires, Department of Immunology, Institut Pasteur, Paris, 75015 France; 3 Present Address: Program in Emerging Infectious Diseases, Duke−National University of Singapore Graduate Medical School, 8 College Road, Singapore, 169857 Singapore; 4 Present Address: Center for Translational Medicine (CTM), International Clinical Research Center (ICRC), St. Anne’s University Hospital, Pekarska 53, 656 91 Brno, Czech Republic; 5 Present Address: Kwazulu-Natal Research Institute for Tuberculosis and HIV, Nelson R. Mandela Medical School, 719 Umbilo Rd, Durban, 4001 South Africa; 6 Present Address: Cancer Science Institute of Singapore (CSI), National University of Singapore (NUS), 14 Medical Drive, Singapore, 117599 Singapore; 7 Present Address: Toscana Life Science Foundation, Via Fiorentina 1, 53100 Siena, Italy; 8 Present Address: San Raffaele Telethon Institute for Gene Therapy (SR-Tiget), IRCCS San Raffaele Scientific Institute, Via Olgettina 58, 20132 Milan, Italy

## Abstract

The intestinal immune system can respond to invading pathogens yet maintain immune tolerance to self-antigens and microbiota. Myeloid cells are central to these processes, but the signaling pathways that underlie tolerance versus inflammation are unclear. Here we show that mice lacking Calcineurin B in CD11c^high^MHCII^+^ cells (*Cnb1*^*CD11c*^ mice) spontaneously develop intestinal inflammation and are susceptible to induced colitis. In these mice, colitis is associated with expansion of T helper type 1 (Th1) and Th17 cell populations and a decrease in the number of FoxP3^+^ regulatory T (Treg) cells, and the pathology is linked to the inability of intestinal *Cnb1*-deficient CD11c^high^MHCII^+^ cells to express IL-2. Deleting IL-2 in CD11c^high^MHCII^+^ cells induces spontaneous colitis resembling human inflammatory bowel disease. Our findings identify that the calcineurin–NFAT–IL-2 pathway in myeloid cells is a critical regulator of intestinal homeostasis by influencing the balance of inflammatory and regulatory responses in the mouse intestine.

## Abstract (web summary)

Treg cells can maintain intestinal homeostasis and limit intestinal bowel disease. Here the authors use a mouse model of spontaneous colitis to show that calcineurin-NFAT-induced IL-2 production by dendritic cells regulates the balance between Treg and effector T cells in the gut lamina propria.

## Introduction

The intestine is a unique and challenging immune microenvironment in which homeostasis requires active tolerance towards self-antigens, dietary-antigens, and commensal microorganisms, balanced with the requirement to detect and respond rapidly to pathogen invasion. Dysregulation of the intestinal immune compartment can result in inflammatory bowel disease (IBD), which affects ~3.5 million people in the US and Europe^1^.

The mouse intestinal lamina propria (LP) contains distinct subsets of mononuclear phagocytes that maintain tolerance and immune reactivity towards pathogens^2,3^. These subsets include conventional dendritic cells (DC), monocytes, and tissue-resident macrophages. LP DCs express high levels of CD11c, MHCII, and FMS-like tyrosine kinase 3 (FLT3, also known as CD135) and FLT3 ligand (FLT3L), and are negative for the lineage markers CD3, CD19, B220, NK1.1, and CD64, whereas the monocytes and macrophages predominantly express CD64 and CD11b^3^. Intestinal DCs comprise three major subsets divided by CD103 and CD11b expression: CD103^+^CD11b^−^, CD103^+^CD11b^+^, and CD103^−^CD11b^+^. Similar to LP populations, these DCs are present in the mesenteric lymph nodes (MLN), suggesting that they contribute to initiation of immune responses. Despite the importance of these myeloid cells in supporting intestinal immune homeostasis, we know little about the cellular signaling networks that modulate tolerance-versus-inflammation in the intestine.

The nuclear factor of activated T-cells (NFAT) family of transcription factors is an important mediator of DC function^4,5^. Initially, NFAT members were thought to be involved solely in the transcriptional activation of T cells^6^, but different NFAT proteins are now known to have a complex influence on host tolerance to self and foreign antigens^7–9^. Upon calcium influx induced by ligation of pattern recognition receptors, activated NFAT triggers calmodulin-mediated activation of the protein phosphatase calcineurin, which in turn induces NFAT nuclear translocation^4,10^.

NFAT is a master transcription factor that regulates expression of IL-2, an immuno-regulatory cytokine that controls T-cell expansion and differentiation, Treg-cell maintenance, and NK-cell activation^6^. DCs are a source of IL-2^11,12^ and IL-2 can regulate immune responses in the mouse lung during fungal infection^5,13^, and promote adaptive immune responses to alum immunization^14^. Whether the calcineurin–NFAT–IL-2 axis in antigen-presenting cells (APC) can contribute to immune homeostasis under basal and inflammatory conditions in other organs, such as the intestine, is unknown.

Here we use mice lacking calcineurin B (encoded by *Cnb1*) or IL-2 (encoded by *Il2*) in CD11c^high^MHCII^+^ cells, and show that calcineurin–NFAT–IL-2 signaling in intestinal CD11c^high^MHCII^+^ APCs prevents spontaneous chronic intestinal inflammation in acute and chronic models of induced colitis. Calcineurin–NFAT signaling in intestinal DCs mediates IL-2 production, which supports local Treg cell expansion in the intestinal LP and restrains inflammatory T-cell expansion and effector function. Our data shed light on the processes underpinning tolerance versus inflammation in the mouse intestine, and identify the calcineurin–NFAT–IL-2 pathway in intestinal APCs as a potential therapeutic target for IBD.

## Results

### Calcineurin–NFAT is active in colonic CD11c^high^MHCII^+^ cells

**Fig. 1:**
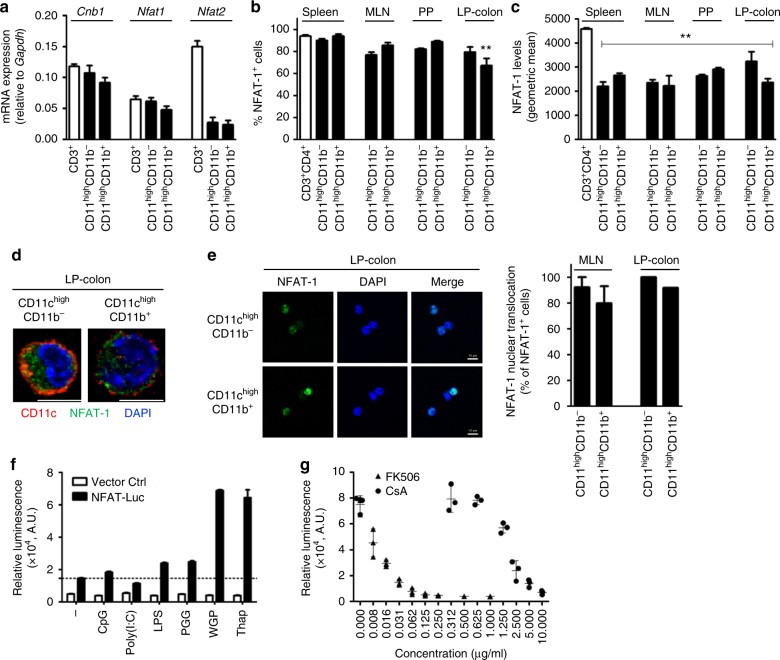
Calcineurin B and NFAT expression in mouse intestinal myeloid cells. **a** Relative expression levels of *Cnb1*, *Nfat1*, and *Nfat2* mRNAs in intestinal CD11c^high^MHCII^+^ cells (CD11b^+^ and CD11b^−^) and MLN CD3^+^ T cells, assessed by qRT-PCR. Data represent the means ± standard error of three experiments (*n* ≥ 7 mice/exp). **b**, **c** Percentage of NFAT-1^+^ cells (**b**) and NFAT-1 protein levels (**c**) in CD11c^high^MHCII^+^ cells (CD11b^+^ and CD11b^−^) obtained from spleen, MLN, PP, and colonic LP (LP-colon), assessed by flow cytometry. Splenic CD3^+^CD4^+^ T cells are included for comparison. Data represent the means ± standard error of three experiments (*n* = 4–5 mice/experiment). **P < 0.01 versus CD4^+^ T cells (ANOVA followed by Dunnett’s multiple comparisons test). **d** Representative images of NFAT-1 labeling in sorted colonic CD11c^high^MHCII^+^ cells (CD11b^+^ and CD11b^−^). Scale bar 5 μm. **e** NFAT-1 nuclear translocation in CD11c^high^MHCII^+^ cells (CD11b^+^ and CD11b^−^) after 30 min stimulation with thapsigargin (Thap; 200 nm), as assessed by confocal analysis. Data represent the means ± standard error of two experiments (*n* = 6–10 mice/experiment). Scale bar 5 μm. **f** NFAT-dependent luciferase activity measured in a DC cell line in response to TLRs (Poly I:C, LPS), dectin-1 ligands (PGG and WGP), and the calcium mobilizer thapsigargin. **g** Dose-dependent inhibition of NFAT nuclear translocation by cyclosporin A (CsA) and tacrolimus (FK506) in WGP-stimulated D1 cells, as assessed by NFAT-luciferase activity. AU arbitrary units, Ctrl control, Luc luciferase, LP lamina propria, LPS Lipopolysaccharide, MLN mesenteric lymph node, PGG soluble β-(1,3)-glucan, PP Peyer’s Patches, WGP whole glucan particles, Thap thapsigargin

mRNA expression analysis of *Cnb1* and the major *Nfat* isoforms in leukocytes *Nfat1* and *Nfat2* revealed that T cells from mesenteric lymph nodes (MLN) and CD11b^–^ and CD11b^+^ CD11c^high^MHCII^+^ cells from colonic lamina propria (LP-colon) expressed *Cnb1* and *Nfat1* at comparable levels, but *Nfat2* was less abundant in both populations compared to T cells (Fig. 1a). At the protein level, NFAT-1 was expressed by ~80% of CD11c^high^CD11b^–^ and CD11c^high^CD11b^+^ myeloid cells of the spleen, MLN, Peyer’s patches (PP) and LP-colon and spleen CD3^+^CD4^+^ T cells **(**Fig. 1b). Fewer LP-colon CD11c^high^CD11b^+^ cells expressed NFAT-1 (~70%). Despite comparable frequencies of NFAT-1^+^ cells, the mean fluorescence intensity of NFAT-1 in both myeloid cell populations was significantly lower than in colonic CD3^+^CD4^+^ T cells (Fig. 1c). Cytoplasmic NFAT-1 in LP-colon CD11c^high^CD11b^–^ and CD11c^high^CD11b^+^ cells was confirmed by confocal microscopy (Fig. 1d) and its translocation to the nucleus was observed in response to the calcium mobilizer thapsigargin (Fig. 1e). Using a mouse DC line (D1) stably expressing an NFAT-luciferase reporter, we found that whole-glucan particles (WGP; particulated dectin-1 agonist) and thapsigargin could robustly activate NFAT; lipopolysaccharide (LPS) and soluble β-(1,3)-glucan PGG activated NFAT to a lesser extent (Fig. 1f). Finally, inhibition of calcineurin signaling with cyclosporin A or tacrolimus (FK506) effectively suppressed WGP-induced NFAT-luciferase activity (Fig. 1g). These data indicate that the calcineurin–NFAT pathway is active under steady-state conditions in colonic CD11c^high^MHCII^+^ cells, and can respond to calcium flux and TLR/dectin-1 ligands.

### Calcineurin B in myeloid cells maintains intestinal homeostasis

**Fig. 2:**
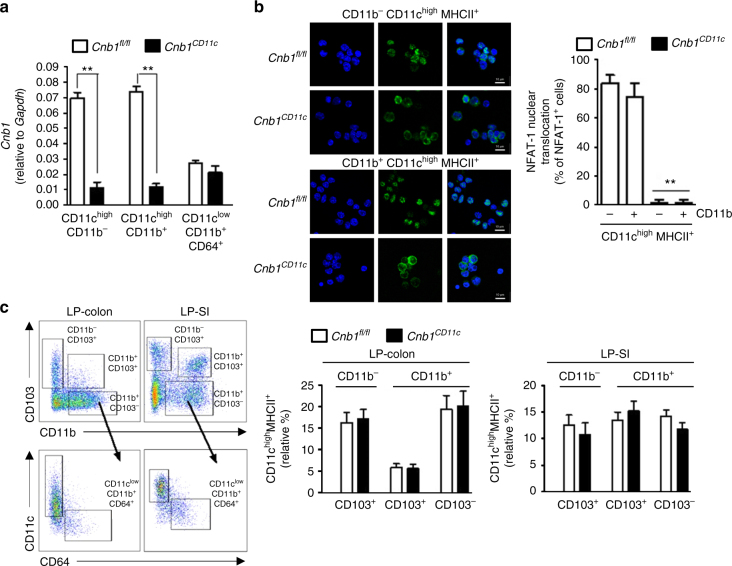
*Cnb1* deletion in CD11c^high^MHCII^+^ cells abrogates NFAT-1 nuclear translocation. **a** *Cnb1* mRNA levels in DCs (CD11c^high^MHCII^+^CD11b^+^, CD11c^high^MHCII^+^CD11b^−^) and macrophages (CD11c^low^MHCII^+^CD11b^+^CD64^+^) isolated from the LP-colon of *Cnb1*^*CD11c*^ and *Cnb1*^*fl*/*fl*^ mice, measured by qRT-PCR. Data represent the means ± standard error of three experiments (*n* = 3 mice/experiment). **P < 0.01. **b** Confocal microscopy analysis of NFAT-1 nuclear translocation in CD11c^high^MHCII^+^ cells (CD11b^+^ and CD11b^−^) from the MLN of *Cnb1*^*CD11c*^ and *Cnb1*^*fl*/*fl*^ mice after thapsigargin stimulation for 30 min. Data represent the means ± standard error of two experiments (*n* = 3 mice/experiment). **P < 0.01. Scale bar 10 μm. **c** Representative flow cytometric analysis of intestinal LP mononuclear myeloid CD11c^+^ cells evaluated by CD11c, CD103, CD11b, and CD64 expression. Four myeloid cell subsets were identified: three DC populations, CD11c^high^CD103^+^, CD11c^high^CD103^+^CD11b^+^, CD11c^high^CD103^−^CD11b^+^ and one CD11c^low^CD103^−^CD11b^+^ macrophage population. The macrophage population was identified based on the expression of CD64 within the CD11c^low^CD103^−^CD11b^+^ population (CD11c^low^ CD64^+^). The frequency of the four different myeloid subsets in LP-colon (left) and LP-SI (right) of *Cnb1*^*CD11c*^ and *Cnb1*^*fl*/*fl*^ mice is shown. Data represent the means ± standard error of three experiments (*n* = 2–3 mice/group per experiment, aged 8–12 weeks). LP lamina propria, SI small intestine

We next asked whether the calcineurin–NFAT pathway has a functional role in intestinal immune homeostasis. We generated *Cnb1*^*CD11c*^ mice in which calcineurin B expression is lost in cells expressing CD11c at high level, by crossing *Cnb1*^*fl*/*fl*^ mice with CD11c-specific Cre-mice^15^. *Cnb1* mRNA was significantly diminished in LP-colon of CD11c^high^MHCII^+^CD11b^−^ and CD11c^high^MHCII^+^CD11b^+^ myeloid cells (mostly belonging to the DC pool) of *Cnb1*^*CD11c*^ mice, compared to CD11c^low^CD11b^+^CD64^+^ cells (mostly macrophages) (Fig. 2a). *Cnb1* deletion prevented thapsigargin-driven NFAT-1 nuclear translocation in CD11b^+^ and CD11b^−^ CD11c^high^MHCII^+^ cell populations in the MLN (Fig. 2b). However, calcineurin–NFAT-1 deficiency in CD11c^high^MHCII^+^ cells did not affect the relative abundance of these myeloid populations in LP-colon of *Cnb1*^*CD11c*^ mice (Fig. 2c), or the expression of maturation markers of CD11c^high^MHCII^+^ myeloid cells in LP-colon and LP-small intestine (LP-SI) (Supplementary Fig. 1). Calcineurin B expression remained intact in B, NK, and mast cells, and naïve, memory CD4^+^ T cells and Treg cells from MLN of *Cnb1*^*CD11c*^ mice, which also released normal cytokine levels in vitro (Supplementary Fig. 2a–c).

To confirm that *Cnb1* deletion in CD11c^high^MHCII^+^ cells did not impact T-cell function in vivo, we monitored the development of colitis following adoptive transfer of naïve CD4^+^CD45RB^high^CD25^−^ T cells from *Cnb1*^*CD11c*^ and *Cnb1*^*fl*/*fl*^ mice into immune-deficient *Rag2*^*KO*^ mice^16^. A normal course of colitis development was observed in *Rag2*^*KO*^ mice receiving naïve CD4^+^ T cells from *Cnb1*^*CD11c*^ mice (Supplementary Fig. 2d, e). These data indicate that CD11c^high^MHCII^+^ cells are the predominant cell population targeted by the *Cnb1* deletion and the abundance and activation status of myeloid cells and effector function of CD4^+^ T cells in the intestine of *Cnb1*^*CD11c*^ mice remain unaffected.

**Fig. 3:**
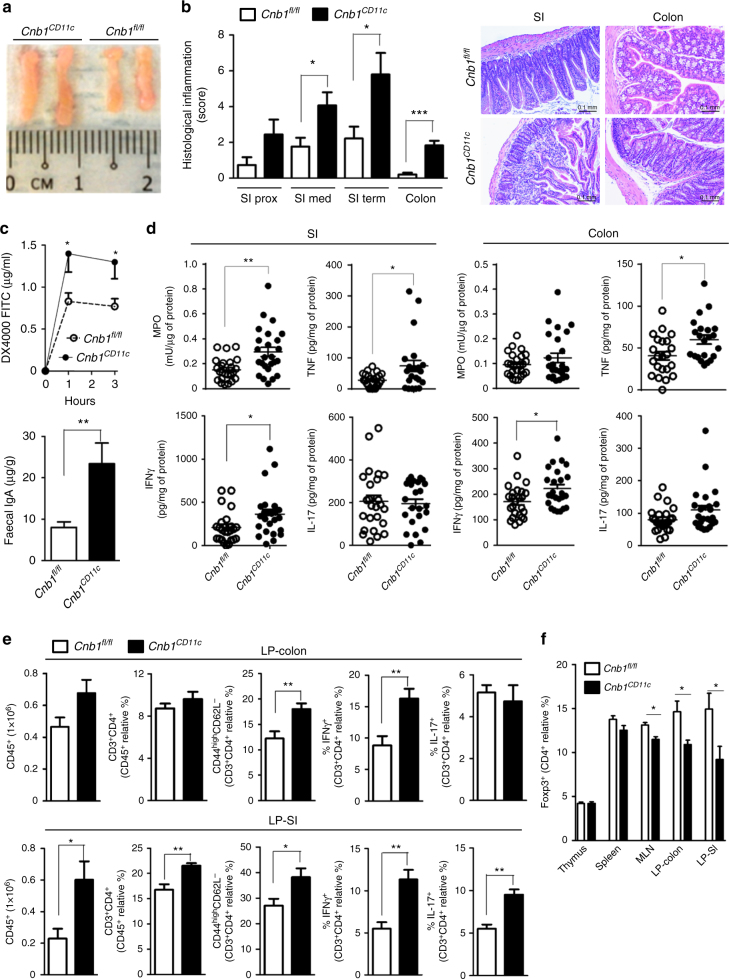
*Cnb1*^*CD11c*^ mice exhibit high intestinal permeability and inflammation. **a** MLN from *Cnb1*^*CD11c*^ and *Cnb1*^*fl*/*fl*^ mice. **b** Histological inflammation index of proximal (prox; *n* = 15–20 mice), medial (med; *n* = 22–27 mice), and terminal (term; *n* = 20–25 mice) small intestine (SI) and colon (*n* = 24–29 mice) in *Cnb1*^*CD11c*^ and *Cnb1*^*fl*/*fl*^ mice. *P < 0.05, ***P < 0.0001. Representative images of H&E-stained terminal ileum (SI) and colon sections from *Cnb1*^*CD11c*^ and *Cnb1*^*fl*/*fl*^ mice (×10 magnification). Scale bar 0.1 mm. **c** Intestinal permeability of *Cnb1*^*CD11c*^ and *Cnb1*^*fl*/*fl*^ mice was assessed by measuring the FITC-dextran concentration in the plasma after FITC-dextran administration by oral gavage (upper panel). *P < 0.05. IgA levels in the fecal content of colons from *Cnb1*^*CD11c*^ and *Cnb1*^*fl*/*fl*^ mice (lower panel). **P < 0.01. Data shown are from three independent experiments (*n* = 5–8 mice/group per experiment). **d** Myeloperoxidase, TNF, IFNγ, and IL-17 levels in terminal SI and colon tissue homogenates from *Cnb1*^*CD11c*^ and *Cnb1*^*fl*/*fl*^ mice, assessed by ELISA. Data shown are from four experiments (*n* = 5–6 mice/group per experiment). *P < 0.05, **P < 0.01. **e** Leukocyte abundance and population composition in the SI and colonic LP of *Cnb1*^*CD11c*^ and *Cnb1*^*fl*/*fl*^ mice. Data represent the mean number of CD45^+^ LP mononuclear cells obtained from each colon or SI, the total percentage of CD4^+^ T cells, the percentage of antigen-experienced CD4^+^ T cells (CD44^high^CD62L^neg^), and of CD4^+^ T cells producing IL-17 or IFNγ. Data represent the means ± standard error of 7–9 experiments (*n* = 5–7 mice/group per experiment). *P < 0.05, **P < 0.01. **f** Percentage of FoxP3^+^ CD4^+^ T cells in the thymus, spleen, MLN, LP-colon, and LP-SI of *Cnb1*^*CD11c*^ and *Cnb1*^*fl*/*fl*^ mice. Data represent the means ± standard error of 3–6 experiments (*n* = 3–6 mice/group). *P < 0.05. DX4000 4 kDa dextran, FITC fluorescein isothiocyanate, LP lamina propria, MLN mesenteric lymph node, MPO myeloperoxidase

Having confirmed the specificity of our *Cnb1* deletion to CD11c^high^MHCII^+^ cells, we asked whether disruption of calcineurin–NFAT signaling in these cells affected intestinal homeostasis in vivo. Macroscopic examination of *Cnb1*^*CD11c*^ mice (aged 10–14 weeks) revealed moderate enlargement of the MLN compared to *Cnb1*^*fl*/*fl*^ control mice (Fig. 3a), and spontaneous inflammation of the SI and colon, characterized by an inflammatory infiltrate in the submucosa with marked erosion of the mucosal lining (Fig. 3b). Although *Cnb1*^*CD11c*^ mice did not develop evident symptoms of colitis (such as weight loss, diarrhea or rectal bleeding), they exhibited significantly higher intestinal permeability, titers of fecal IgA (Fig. 3c), and myeloperoxidase activity in homogenates of the terminal ileum (Fig. 3d) than *Cnb1*^*fl*/*fl*^ controls. Increased levels of mucosal TNF and IFNγ were evident in the SI and colon of *Cnb1*^*CD11c*^ mice, compared to *Cnb1*^*fl*/*fl*^ controls, while IL-17 levels were comparable (Fig. 3d).

Analysis of the immune-cell composition and cytokine profile in the *Cnb1*^*CD11c*^ LP revealed a higher frequency of immune cells (CD45^+^ and CD4^+^ T cells), and a higher percentage of antigen-experienced CD44^+^CD62L^−^ CD4^+^ T cells in the LP-SI and LP-colon compared to *Cnb1*^*fl*/*fl*^ controls (Fig. 3e). Cnb1 deficiency in CD11c^high^MHCII^+^ myeloid cells also significantly increased the frequency of IL-17-producing and IFNγ-producing CD4^+^ T cells infiltrating the LP-SI, and IFNγ-producing CD4^+^ T cells in the LP-colon (Fig. 3e). No difference in IL-4-producing CD4^+^ T cells or the CD8^+^ T-cell phenotype was observed in the LP-colon of *Cnb1*^*CD11c*^ mice compared to *Cnb1*^*fl*/*fl*^ controls (Supplementary Fig. 3a). Importantly, *Cnb1*^*CD11c*^ mice exhibited significantly reduced frequencies of FoxP3^+^ Treg cells in the LP-colon, LP-SI, and MLN, but not in the spleen or thymus (Fig. 3f). Spontaneous chronic inflammation occurred exclusively in the intestine and systemic inflammation, immune phenotype and cytokine release from splenic CD4^+^ T cells were comparable between *Cnb1*^*CD11c*^ and *Cnb1*^*fl*/*fl*^ mice (Supplementary Fig. 3b, c).

These data support that the calcineurin–NFAT axis in CD11c^high^MHCII^+^ cells is critical for CD4^+^ T-cell homeostasis in the intestine under steady-state conditions.

### *Cnb1*^*CD11c*^ mice have unrestrained induced gut inflammation

**Fig. 4:**
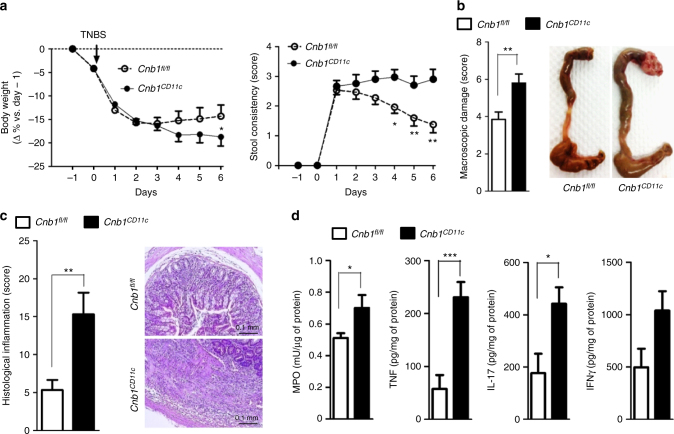
Exacerbation of acute and chronic colitis in *Cnb1*^*CD11c*^ mice. **a** Severity of TNBS-induced colitis evaluated according to weight loss (left panel) and stool consistency (right panel) in *Cnb1*^*CD11c*^ and *Cnb1*^*fl*/*fl*^ mice. **b** Macroscopic index (left) and overt anatomy of the colon (right) and **c** histological inflammation score for colons of *Cnb1*^*CD11c*^ and *Cnb1*^*fl*/*fl*^ mice after TNBS administration. Representative H&E-stained colon sections showing infiltrating leukocytes (×10 magnification, scale bar 0.1 mm). **d** Levels of inflammatory markers (MPO, TNF, IL-17, and IFNγ) in total colon homogenates from *Cnb1*^*CD11c*^ and *Cnb1*^*fl*/*fl*^ mice. All data represent the means ± standard error of two experiments (*n* = 6–7 mice/group per experiment). *P < 0.05, ***P < 0.001. TNBS trinitrobenzenesulfonic acid, MPO myeloperoxidase

We next asked how dysregulated T-cell responses would affect the ability of *Cnb1*^*CD11c*^ mice to restrain pathologic mucosal inflammation during acute and chronic models of IBD. Intra-rectal instillation of trinitrobenzene sulfonate (TNBS) rapidly induces intestinal inflammation in mice that recapitulates key features of Crohn’s disease^17,18^. Compared to TNBS-treated *Cnb1*^*fl*/*fl*^ controls, TNBS-treated *Cnb1*^*CD11c*^ mice exhibited a significant loss in body weight by 6 days post treatment, worse stool consistency (Fig. 4a), and severe ulceration and thickening of the colon wall (Fig. 4b). Using a histopathological scoring approach, we found that *Cnb1*^*CD11c*^ mice exhibited severe leukocyte infiltration that penetrated all colon layers (Fig. 4c) and a significant increase in myeloperoxidase activity, TNF, and IL-17 in colon homogenates (Fig. 4d). Treatment of *Cnb1*^*CD11c*^ mice with naproxen, a non-steroidal anti-inflammatory drug that can exacerbate colitis in susceptible IBD patients, also induced notable colonic mucosal inflammation compared to untreated *Cnb1*^*CD11c*^ mice or naproxen-treated *Cnb1*^*fl*/*fl*^ control mice (Supplementary Fig. 4). These results show that calcineurin B expression in CD11c^high^MHCII^+^ cells not only prevents spontaneous colitis under steady-state conditions, but also helps suppress severe intestinal pathology in response to acute and chronic inflammation.

### *Cnb1* deletion in non-DC phagocytes reduces colitis severity

To examine the effect of *Cnb1* depletion in monocytes, macrophages, and granulocytes on intestinal inflammation, we generated a *Cnb1*^*LysM*^ mouse model, in which the *Cnb1* floxed allele was deleted by Cre-recombinase in cells expressing LysM^19^. The intestinal phenotype of LP mononuclear cells from *Cnb1*^*LysM*^ mice under steady-state conditions was indistinguishable from *Cnb1*^*fl*/*fl*^ controls with respect to: maintenance of body weight, colon histology, LP-colon immune-cell phenotypes, and levels of pro-inflammatory cytokines (Supplementary Fig. 5a–c). Moreover, upon TNBS treatment, symptoms of acute colitis were significantly less severe in *Cnb1*^*LysM*^ mice than in *Cnb1*^*fl*/*fl*^ controls (Supplementary Fig. 5d–f).

These data highlight that calcineurin B expression in CD11c^high^MHCII^+^LysM^−^ cells, which mainly represent the conventional DCs in the intestine, participates in intestinal homeostasis by restraining pathological inflammation during colitis while calcineurin B expressed in LysM^+^ monocytes/macrophages and granulocytes has little effect on intestinal homeostasis during steady-state conditions, but exacerbates intestinal immunopathology during TNBS-induced colitis.

### Calcineurin B partially regulates DC-derived IL-2 production

**Fig. 5:**
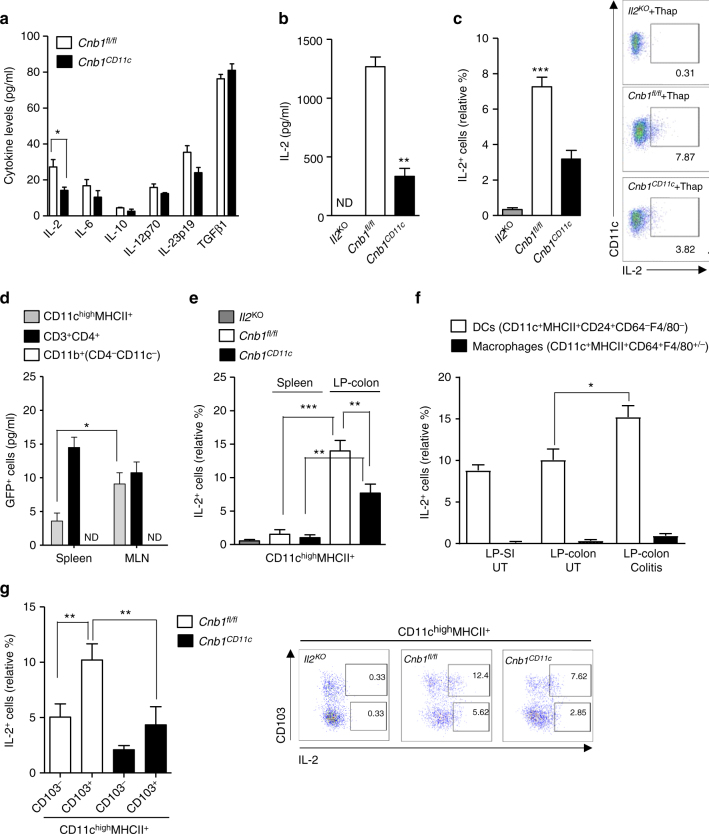
Calcineurin-NFAT signaling in CD11c^+^MHCII^high^ myeloid cells regulates IL-2 synthesis. **a** CD11c^high^MHCII^high^CD11b^−^ cells sorted from MLN of *Cnb1*^*CD11c*^ and *Cnb1*^*fl*/*fl*^ mice were stimulated with thapsigargin for 16–20 h. Cytokine levels were measured in the culture supernatants using Luminex technology. Data represent the means ± standard error of three experiments (*n* = 10 mice/group per experiment). *P < 0.05. **b**, **c** IL-2 production from bone marrow-derived DCs of *Il2*^*KO*^, *Cnb1*^*CD11c*^, and *Cnb1*^*fl*/*fl*^ mice in response to thapsigargin stimulation for 16 h, as assessed by ELISA (**b**) and by intracellular labeling (**c**). Data represent the means ± standard error of three experiments (*n* = 1–2 mice/group; mice aged 6–7 weeks old). **P < 0.01, ***P < 0.001. **d** GFP (corresponding to IL-2) expression in CD11c^high^MHCII^+^ cells, CD3^+^CD4^+^ T cells, and total myeloid CD11b^+^ (CD4^−^CD11c^−^) cells isolated from the spleen and MLN of IL-2-GFP reporter mice. Data represent the means ± standard error of four experiments (*n* ≥ 3 mice/group). *P < 0.05. **e** Intracellular IL-2 labeling of colonic CD3^−^CD11c^high^MHCII^+^ cells from *Il2*^*KO*^, *Cnb1*^*fl*/*fl*^ and *Cnb1*^*CD11c*^ mice. Data are presented as the relative percentage of IL-2^+^ cells and represent the means ± standard error of five experiments (*n* = 3–4 mice/group per experiment). **P < 0.01, ***P < 0.001. **f** Percentage of IL-2^+^ cells in intestinal DC (CD45^+^Lin^−^CD3^−^CD11c^+^MHCII^+^CD24^+^CD64^−^F4/80^−^) and macrophage (CD45^+^Lin^−^CD3^−^CD11c^+^MHCII^+^CD24^−^CD64^+^F4/80^+/−^) populations in LP-colon and LP-small intestine of *Cnb1*^*fl*/*fl*^ mice at steady state, or during colitis induced in immunocompromised *Rag1*^*KO*^ mice by adoptive transfer of naïve CD4^+^ T cells (CD45RB^high^CD62L^+^CD44^−^CD25^−^) isolated from the spleens of C57BL/6 mice. Data represent the means ± standard error of three experiments (5–6 mice/group). **g** IL-2 expression in CD103^−^ and CD103^+^ CD11c^high^MHCII^+^ cells from colons of *Cnb1*^*fl*/*fl*^ and *Cnb1*^*CD11c*^ mice. Representative dot plots of IL-2 labeling in colonic CD3^−^CD11c^high^MHCII^+^ cells are shown. Data represent the means ± standard error of three experiments (*n* = 2–5 mice/group per experiment). *P < 0.05. KO knockout, LP lamina propria, MLN mesenteric lymph node, SI small intestine, Thap thapsigargin, UT untreated, ND not detected

To delineate the mechanistic basis for calcineurin-mediated protection from colitis elicited by CD11c^high^MHCII^+^ DCs, we examined a role for calcineurin B in the production of DC cytokines regulating T-helper differentiation. Colonic CD11c^high^MHCII^+^CD11b^−^ cells from *Cnb1*^*CD11c*^ mice produced significantly less IL-2 following thapsigargin exposure compared to cells from *Cnb1*^*fl*/*fl*^ mice, while the levels of IL-6, IL-12, IL-23, IL-10, and TGFβ were comparable (Fig. 5a). Similarly, we found that bone marrow (BM)-derived DCs released IL-2 in response to thapsigargin, LPS, and zymosan, as assessed by ELISA and intracellular IL-2 labeling (Fig. 5b, c and Supplementary Fig. 6a), whereas IL-2 production from *Cnb1*^*CD11c*^ DCs, but not IL-12p40 or IL-6, was significantly reduced. Moreover, exposure to NFκB and p38 MAPK inhibitors fully abrogated IL-2 release from *Cnb1*^*CD11c*^ DCs (Supplementary Fig. 6b). These data indicate that IL-2 synthesis in DCs depends on calcineurin–NFAT, NFκB and AP1 signaling.

We also evaluated the ability of CD11c^high^MHCII^+^ cells to produce IL-2 in vivo using an IL-2–GFP reporter mouse^20^. GFP^+^ (IL-2^+^) cells among the CD11c^high^MHCII^+^ compartment were present in the spleen (~2–4%), MLN (~8–10%), and were absent in CD11b^+^ (CD4^−^CD11c^−^) cells belonging to the monocyte/macrophage/granulocyte lineage (Fig. 5d and Supplementary Fig. 7). Intracellular IL-2 labeling of CD11c^high^MHCII^+^ cells isolated from spleen and LP-colon showed that up to ~13% of cells produced IL-2 under steady-state conditions (Fig. 5e and Supplementary Fig. 8). During acute colitis, the proportion of IL-2-producing CD11c^+^MHCII^+^ (CD64^−^F4/80^−^) DCs in LP-colon further increased (Fig. 5f and Supplementary Fig. 9a, b). Moreover, intestinal macrophage population (CD11c^+^MHCII^+^CD64^+^F4/80^+/−^) was negative for IL-2 labeling both during steady-state and colitic conditions (Fig. 5f and Supplementary Fig. 9a, b). IL-2 production by CD11c^+^MHCII^+^ DCs, but not macrophages, was also confirmed by qRT-PCR (Supplementary Fig. 9c). These data indicate that IL-2 expression in vivo is high in the intestine and restricted to the CD11c^+^MHCII^+^ DC population (predominantly CD103^+^ cells) (Fig. 5g and Supplementary Fig. 9).

To examine whether deficient IL-2 expression in CD11c^high^MHCII^+^ DCs of *Cnb1*^*CD11c*^ mice was the key factor involved in dysregulation of intestinal immune responses, we administered IL-2:anti-IL-2 complexes (IL-2C), which increase IL-2 bioactivity in vivo, and assessed whether normal intestinal homeostasis could be restored in these mice. While the proportions of Foxp3^+^ Treg cells were increased to a similar extent in the spleens of both *Cnb1*^*CD11c*^ and *Cnb1*^*fl*/*fl*^ mice after IL-2C treatment, these cells expanded only in the intestinal mucosa of *Cnb1*^*CD11c*^ mice compared to *Cnb1*^*fl*/*fl*^ controls, suggesting that lack of *Cnb1* in CD11c^+^ cells is partly responsible for altered numbers of Treg cells in these mice (Supplementary Fig. 10a). To confirm that the impairment in DC-derived IL-2 signaling induces intestinal CD4^+^ T-cell dysregulation observed in *Cnb1*^*CD11c*^ mice, we adoptively transferred wild-type or *Il2*-null DCs into the *Cnb1*^*CD11c*^ mice. Wild-type DCs selectively restored the levels of activated CD4^+^ T cells producing IFNγ in the LP-colon of *Cnb1*^*CD11c*^ mice compared to untreated or *Cnb1*^*CD11c*^ mice receiving *Il2*-null DCs (Supplementary Fig. 10b). Moreover, wild-type DC transfer into *Cnb1*^*CD11c*^ mice normalized the percentage of Treg cells in MLN, whereas the injection of *Il2*-null DCs induced an additional reduction of colonic FoxP3^+^ Treg cells. (Supplementary Fig. 10c).

### DC IL-2 production prevents severe spontaneous chronic colitis

**Fig. 6:**
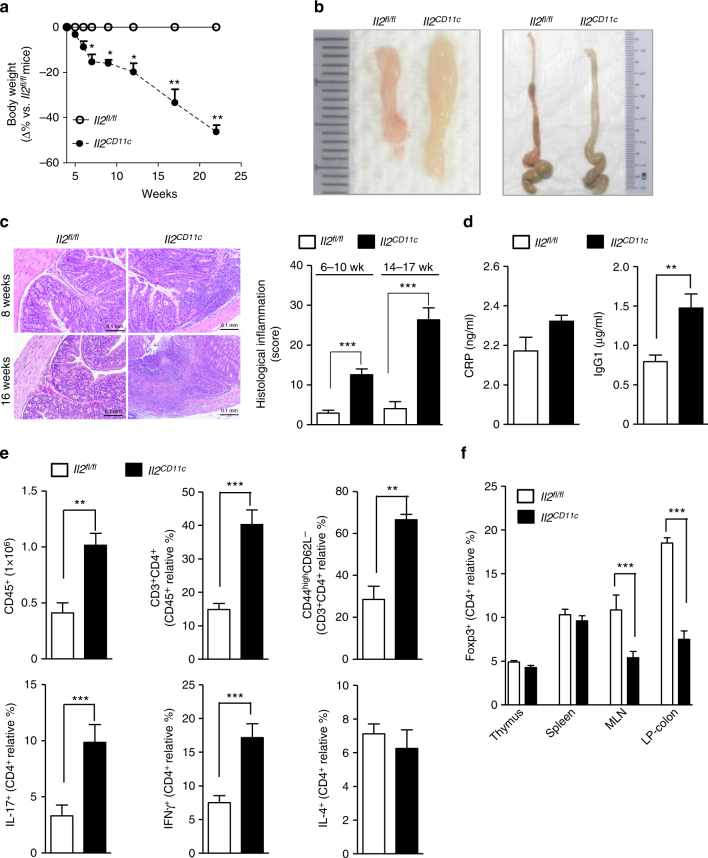
Th1/Th17-cell expansion and Treg-cell contraction in the gut of *Il2*^*CD11c*^ mice. **a** Change in body weight (expressed as Δ percentage relative to the mean weight of the control group) in *Il2*^*CD11c*^ mice compared to *Il2*^*fl*/*fl*^ mice. Data represent the means ± standard error (*n* = 20 mice/group). *P < 0.05, **P < 0.01. **b** Representative images of the MLN (left) and colons (right) from *Il2*^*CD11c*^ and *Il2*^*fl*/*fl*^ mice aged 10 weeks old. **c** H&E staining of colon sections showing massive leukocyte infiltration in *Il2*^*CD11c*^ compared to *Il2*^*fl*/*fl*^ mice (×10 magnification, scale bar 0.1 mm). Histological inflammation index of the colon was also evaluated over time. Data represent the means ± standard error (14 mice/group aged 6–10 weeks old, and 6 mice/group aged 14–17 weeks old). ***P < 0.001. **d** Plasma levels of C-reactive protein (CRP) and IgG1 in the sera of *Il2*^*CD11c*^ and *Il2*^*fl*/*fl*^ mice. Data represent the means ± standard error (5–7 mice/group). **P < 0.01. **e** Immune phenotype of LP-colon mononuclear cells from *Il2*^*CD11c*^ and *Il2*^*fl*/*fl*^ mice. Data represent the means ± standard error of the total number of mononuclear cells obtained from the LP, the percentage of total and antigen-experienced CD44^high^CD62L^-^ CD4^+^ T cells, and the proportion of CD4^+^ T cells producing IL-17, IFNγ or IL-4. **f** Frequency of FoxP3^+^ Treg cells in thymus, spleen, MLN, and LP-colon isolated from *Il2*^*CD11c*^ and *Il2*^*fl*/*fl*^ mice. Data represent the means ± standard error of 3–4 experiments (*n* = 2–4 mice/group per experiment, aged 6–10 weeks). **P < 0.01, ***P < 0.001. LP lamina propria, MLN mesenteric lymph node

A role for T-cell-derived IL-2 in regulating T-cell responses and maintaining Treg-cell-mediated self-tolerance is well-established, but a distinct contribution of myeloid IL-2 to intestinal homeostasis in vivo is unknown. To address this issue, we used an *Il2*^*CD11c*^ knock-out mouse model^5^ to monitor the effects of myeloid *Il2* deficiency. *Il2*^*CD11c*^ mice were initially indistinguishable from *Il2*^*fl*/*fl*^ mice, but from ~5 weeks of age they exhibited significant weight loss, resulting in ~40–45% lower body mass compared to *Il2*^*fl*/*fl*^ mice by 22 weeks (Fig. 6a). Enlargement of the MLN and macroscopic thickening and shortening of the colon without splenomegaly was observed in 7–10-week-old *Il2*^*CD11c*^ mice compared to *Il2*^*fl*/*fl*^ mice (Fig. 6b). Histopathological analysis of colons from 8-week-old *Il2*^*CD11c*^ mice revealed moderate leukocyte infiltration of the mucosa and sub-mucosa, which affected 50–100% of the colon surface (Fig. 6c); this pathology increased in severity with age, until systemic inflammation (high serum levels of CRP and IgG1) had developed by 14–16 weeks (Fig. 6d). Consistently, *Il2*^*CD11c*^ mice also exhibited substantially higher numbers of CD45^+^ leukocytes and total, antigen-experienced, and IFNγ-producing or IL-17-producing CD4^+^ T cells (Fig. 6e), and a markedly smaller population of FoxP3^+^ Treg cells in colons and the MLN (but not thymus and spleen), compared to *Il2*^*fl*/*fl*^ mice (Fig. 6f). MLN CD4^+^ T cells isolated from *Il2*^*CD11c*^ mice robustly expanded with age (Supplementary Fig. 11a) and produced more IFNγ and IL-17, while IL-2 release was normal when cells were re-stimulated ex vivo with antiCD3/CD28 antibodies (Supplementary Fig. 11b).

**Fig. 7:**
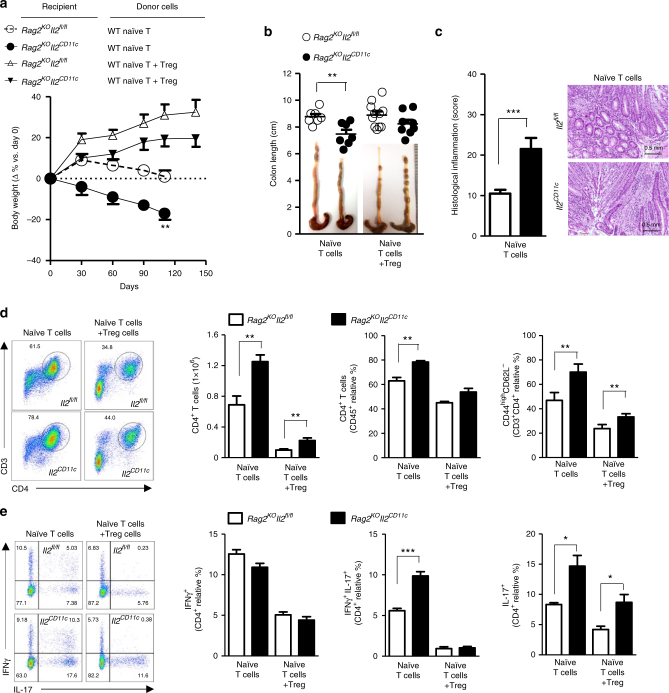
DC-derived IL-2 suppresses pathogenic CD4^+^ T-cell expansion in the intestine. **a** Change in body weight and **b** severity of colitis based on colon length of *Il2*^*CD11c*^ and *Il2*^*fl*/*fl*^, *Rag2* knockout (KO) mice (*Rag2*^*KO*^*IL*-*2*^*fl*/*fl*^ and *Rag2*^*KO*^*IL*-*2*^*CD11c*^ mice) adoptively transferred with naïve CD4^+^ T cells either alone or in combination with Treg cells isolated from spleens of C57BL/6 mice. Representative pictures of colons after adoptive T-cell transfer are shown. Data represent the means ± standard error of two experiments (*n* = 2–5 mice/group per experiment). **P < 0.01. **c** Histological inflammatory score based on H&E staining of colon sections (×10 magnification, scale bar 0.5 mm) obtained from *Rag2*^*KO*^*Il2*^*fl*/*fl*^ and *Rag2*^*KO*^*Il2*^*CD11c*^ mice after 110 days of naïve CD4^+^ T-cell transfer. Data represent the means ± standard error. ***P < 0.001. **d**, **e** Phenotypic analysis of colonic lamina propria CD4^+^ T cells evaluated 110 days after adoptive T-cell transfer. The total number and the relative percentage of total and activated CD4^+^ T cells (**d**), as well as the proportions of CD4^+^ T cells producing IL-17, IFNγ or both (**e**) are shown. *P < 0.05, **P < 0.01, ***P < 0.001. All data represent the means ± standard error of two experiments (*n* = 3–4 mice/group per experiment)

To address the contribution of myeloid IL-2 in LP during differentiation of naïve CD4^+^ T cells in vivo, syngeneic naïve T helper cells from wild-type donor mice were adoptively transferred into *Il2*^*CD11c*^ and *Il2*^*fl*/*fl*^, *Rag*-deficient mice (*Rag2*^*KO*^*Il2*^*CD11c*^ and *Rag2*^*KO*^*Il2*^*fl*/*fl*^, respectively), alone or in combination with wild-type Treg cells^16^. Loss of myeloid IL-2 in the absence of lymphocytes did not affect the LP composition of colonic immune cells (Supplementary Fig. 12). By contrast, *Rag2*^*KO*^*Il2*^*CD11c*^ mice receiving naïve CD4^+^ T cells showed an early and severe onset of wasting disease characterized by substantial weight loss (Fig. 7a), shortening of colon (Fig. 7b), mucosal inflammation (Fig. 7c), and an increase in activated CD4^+^ T cells (Fig. 7d) secreting higher levels of IL-17 and IFNγ (Fig. 7e) compared to *Rag2*^*KO*^*Il2*^*fl*/*fl*^ control mice. Co-transfer of Treg cells prevented colitis by reducing the expansion of activated IFNγ-producing and IL-17-producing CD4^+^ T cells (Fig. 7a–e). After the induction of T-cell-mediated experimental colitis, IL-2 loss in CD11c^high^MHCII^+^ cells was also associated with increased signs of skin and joint inflammation, which are the most common extra-intestinal manifestations of IBD in humans (Supplementary Fig. 13)^21^. These results indicate that a deficiency in myeloid IL-2 is necessary and sufficient to trigger spontaneous autoimmune colitis characterized by spontaneous and chronic mucosal damage reminiscent of Crohn’s disease^22,23^.

**Fig. 8:**
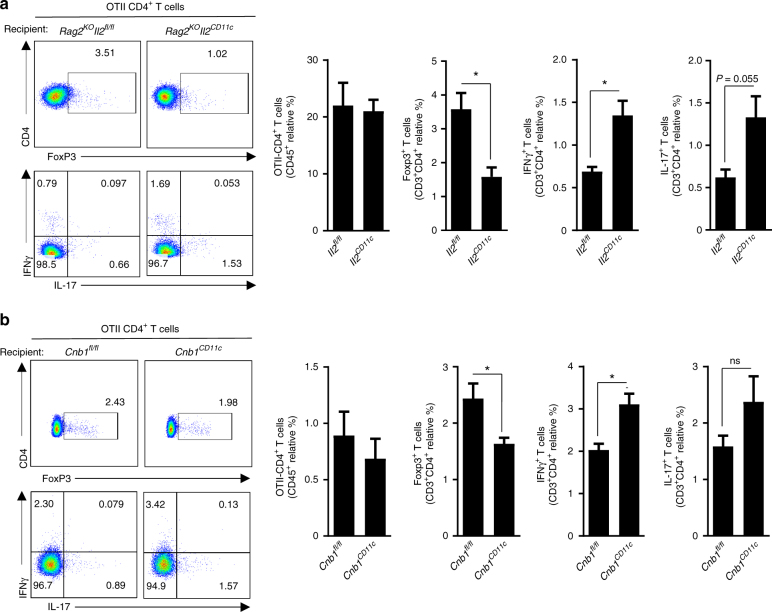
*Il2* or *Cnb1* deficiency in DCs causes a dysregulated T-cell response. **a**,** b** Naïve congenic OTII cells were sorted from spleens of donor mice and transferred intravenously into *Rag2*^*KO*^*Il2*^*fl*/*fl*^ and *Rag2*^*KO*^*Il2*^*CD11c*^ (**a**) or *Cnb1*^*CD11c*^ and *Cnb1*^*fl*/*fl*^ mice (**b**). After 24 h, mice were administered OVA protein antigen by daily oral gavage for 5 days. The frequencies of CD4^+^ T cells expressing FoxP3, IL-17, and IFNγ were examined in the mesenteric lymph node 7 days after adoptive cell transfer. Representative FACS plots of OTII donor CD4^+^ T cells are shown (left panels). Data represent the means ± standard deviation of two experiments (*n* = 1–2 mice/group). *P < 0.05 (two-tailed, unpaired Student’s *t* test)

Treg cells are essential to intestinal homeostasis as they monitor effector T helper-cell activation^17^. As we observed a consistent reduction in Treg cells in both *Cnb1*^*CD11c*^ and *Il2*^*CD11c*^ mice in the MLN and LP, but not in the thymus or spleen (Figs. 3f and 6f), we examined whether myeloid IL-2 is important for the induction of antigen-specific Treg cells in the LP. Naïve CD4^+^ T cells from OTII mice were adoptively transferred into *Rag2*^*KO*^*Il2*^*CD11c*^ and *Rag2*^*KO*^*Il2*^*fl*/*fl*^ mice, and the mice were then fed with ovalbumin (OVA) for 5 days. Conversion of naïve OTII CD4^+^ T cells into FoxP3^+^ Treg cells was significantly impaired, while Th1 differentiation (and to a lesser extent Th17) was significantly enhanced in the MLN of *Rag2*^*KO*^*Il2*^*CD11c*^ mice compared to controls (Fig. 8a); similar results were obtained in *Cnb1*^*CD11c*^ mice (Fig. 8b). These data indicate that myeloid IL-2 regulates Treg cell induction, which can directly suppress effector T helper cell expansion and activity.

### DC and T cell IL-2 have distinct functions in gut homeostasis

**Fig. 9:**
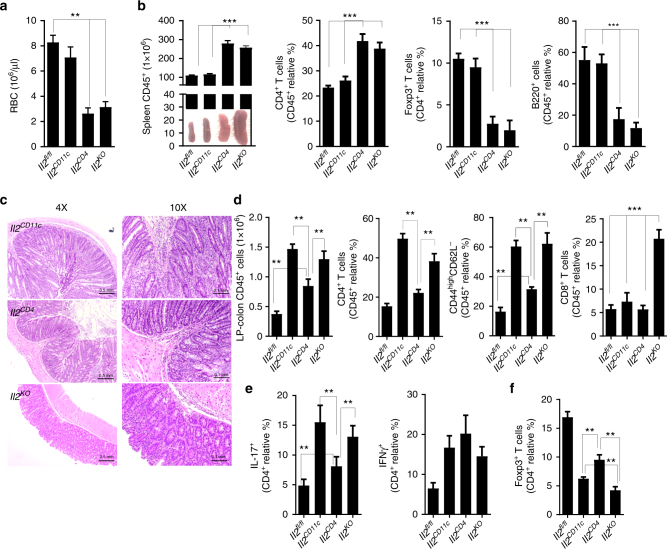
Systemic and intestinal immune homeostasis in *Il2*^*CD11c*^ and *Il2*^*CD4*^ mice. **a** RBC count and **b** total number of splenocytes and proportion of CD4^+^ T cells, FoxP3^+^ Treg cells, and B220^+^ B cells in spleens of *Il2*^*fl*/*fl*^, *Il2*^*CD11c*^, and *Il2*^*CD4*^ mice. Representative images of spleens are shown. Data represent the means ± standard error of 2–6 experiments (*n* = 2–3 mice/group per experiment, 6–13 weeks old). **P < 0.01, ***P < 0.001. **c** Representative images of H&E-stained colon sections (×4 magnification, scale bar 0.5 mm; ×10 magnification, scale bar 0.1 mm) from *Il2*^*CD11c*^, *Il2*^*CD4*^, and *Il2*^*KO*^ mice. **d** Total LP-colon leukocyte number and percentage of total and activated (CD44^high^CD62L^−^) CD4^+^ T cells, and B cells. **e**, **f** Percentage of IFNγ- and IL-17-producing CD4^+^ T cells, and FoxP3^+^ Treg cells obtained from the LP-colon of *Il2*^*fl*/*fl*^, *Il2*^*CD11c*^, *Il2*^*CD4*^, and *Il2*^*KO*^ mice. Data represent the means ± standard error of 2–4 experiments (*n* = 2–3 mice/group per experiment, 9–13 weeks old). **P < 0.01, ***P < 0.001. LP lamina propria, RBC red blood cells

To dissect the contributions of myeloid cell-derived IL-2 and T helper cell-derived IL-2 to intestinal immune homeostasis, we compared the systemic and intestinal phenotypes of *Il2*^*CD11c*^, *Il2*^*CD4*^ (generated by crossing *Il2*^*fl*/*fl*^ and CD4-cre mice), and *Il2*^*KO*^ (carrying IL-2 deletion in myeloid and CD4^+^ T cells) mice. *Il2* deletion in the CD4^+^ T-cell compartment resulted in characteristic IL-2 Deficiency Syndrome^24–26^ originally described in *Il2*^*KO*^ mice and characterized by severe anemia (Fig. 9a), splenomegaly (Fig. 9b), uncontrolled expansion of splenic T cells associated with reduction of Treg cells and loss of B cells. None of these features were observed in *Il2*^*CD11c*^ mice (Fig. 9b), indicating that IL-2 Deficiency Syndrome is mostly driven by the loss of “adaptive” IL-2. The intestinal phenotypes of *Il2*^*CD11c*^ mice were even more divergent from *Il2*^*CD4*^ mice: although both *Il2*^*CD11c*^ and *Il2*^*CD4*^ mice developed colitis at 10–13 weeks, the intestinal pathology was exacerbated in *Il2*^*CD11c*^ mice compared to *Il2*^*CD4*^ mice, and more similar to that of *Il2*^*KO*^ mice (Fig. 9c). A higher increase in total leukocytes, total and activated CD4^+^ T cells, and IL-17-producing CD4^+^ T cells was observed in the LP-colon of *Il2*^*CD11c*^ mice compared to *Il2*^*CD4*^ mice, and was similar to that seen in *Il2*^*KO*^ mice, whereas accumulation of CD8^+^ T cells was specific to *Il2*^*KO*^ mice (Fig. 9d, e). Similar to the LP-colon of *Il2*^*KO*^ mice, frequencies of Treg cells were reduced in the LP-colon of *Il2*^*CD11c*^ and to a lesser extent in *Il2*^*CD4*^ mice compared to *Il2*^*fl*/*fl*^ mice (Fig. 9f). These results indicate that innate (myeloid cells) and adaptive (CD4^+^ T cells) IL-2 exert both distinct and overlapping functions at the systemic level and in the intestine.

## Discussion

This study shows that activation of the calcineurin–NFAT pathway in CD11c^high^MHCII^+^ cells (representing the intestinal DC pool) prevents spontaneous intestinal inflammation in vivo by orchestrating a balance between inflammatory and regulatory responses in the colonic mucosa. By characterizing mice in which *Cnb1* gene was deleted either in CD11c^high^MHCII^+^ APCs (mostly representing the DC pool) but not CD64^+^ macrophages, or LysM-expressing cells (mainly macrophages, granulocytes, and monocytes), we identified two distinct roles for the calcineurin–NFAT pathway: calcineurin activation has an anti-inflammatory function in intestinal CD11c^+^MHCII^high^ DCs, but a pro-inflammatory role in macrophages, granulocytes, and monocytes. Accordingly, *Cnb1*^*CD11c*^ mice developed spontaneous intestinal inflammation, whereas *Cnb1*^*LyzM*^ mice were unaffected by targeted *Cnb1* deletion, and were protected from induced colitis.

We verified that spontaneous chronic colitis in *Cnb1*^*CD11c*^ mice was not due to anomalous clonal composition of the myeloid compartment, as the DC and macrophage populations were normally represented in the LP-colon and no overt differences in co-stimulatory markers expressed by these cells were found in our model. The CD4^+^ T-cell compartment of *Cnb1*^*CD11c*^ mice was also normal: T helper cells (both naïve and memory) and Treg cells expressed normal calcineurin B levels, were able to produce effector cytokines (including IL-2, IFNγ, and IL-10) at normal levels, and naïve T helper cells isolated from these mice were able to induce severe colitis when adoptively transferred into lymphopenic mice.

We also identified the importance of calcineurin–NFAT-mediated production of IL-2 by the intestinal DC pool in regulating intestinal homeostasis. The colonic CD103^+^ DC subset is the main producer of IL-2, whereas CD64^+^F4/80^+/−^ macrophages do not produce this cytokine. Colonic DCs from *Cnb1*^*CD11c*^ mice still retain the ability to release IL-6, IL-12, IL-23. IL-2 deletion in CD11c^high^MHCII^+^ DCs caused more severe, spontaneous intestinal inflammation in *Il2*^*CD11c*^ mice compared to *Cnb1*^*CD11c*^ mice, which was characterized by expansion of activated CD4^+^ effector T cells and substantial reduction of Treg cells. IL-2 complex treatment and DCs transfer results support that reduced levels of DC-derived IL-2 could be the primary cause triggering the dysregulation of intestinal CD4^+^ T cells observed in *Cnb1*^*CD11c*^ mice.

Spontaneous colitis in *Cnb1*^*CD11c*^ mice was milder compared to *Il2*^*CD11c*^ mice, suggesting that other signaling pathways that trigger DC-IL-2 production may exist. Using pharmacological inhibitors, we found that IL-2 release also depends on NFκB in response to LPS, zymosan, and thapsigargin stimulation, and on p38 MAPK in response to zymosan and thapsigargin stimulation. A recent study reported that deletion of TRAF6, a component of the TLR–NFκB pathway, is associated with ~50% reduction of DC-derived IL-2^27^. Our study identifies the calcineurin–NFAT pathway as an additional axis that operates in parallel with the TRAF6–NFκB pathway in myeloid cells to produce IL-2, suggesting that both NFκB and NFAT cooperate to activate IL-2 transcription. Although these pathways contribute to the overall production of IL-2, the nature of the intestinal immune-pathologies observed in the *Traf6*-DC and *Cnb1*-DC-deficient mice diverge: loss of *Traf6* in DCs causes reduced inflammatory cytokines secretion, including IL-12, whereas *Cnb1*-deficient DCs release normal levels of inflammatory cytokines. Moreover, partial loss of IL-2 in *Traf6*-deficient DCs causes spontaneous Th2-mediated enteritis characterized by eosinophilic infiltration and decreased Treg-cell numbers specifically in the small intestine^27^. This phenotype markedly differs from *Cnb1*^*CD11c*^ mice, as these mice develop a spontaneous form of enteritis in both the small and large intestine resembling Crohn’s disease, and is associated with Th1 and Th17 colitogenic T-cell expansion, mucosal accumulation of mononuclear cells and progressive colon thickening^28^. Through a series of in vivo experiments, our data suggest that the calcineurin–NFAT–IL-2 pathway in myeloid cells is crucial for the induction of Treg cells, which contributes to maintaining immune tolerance in the gut by preventing autoimmunity. Indeed, *Il2*^*CD11c*^ and lymphopenic *Rag2*^*KO*^*Il2*^*CD11c*^ mice receiving normal naïve T cells developed an exacerbated form of spontaneous colitis associated with extra-intestinal manifestations, including cutaneous and articular inflammatory reactions, which are the foremost comorbidities of IBD^21^.

To date, the regulatory and effector functions of the calcineurin–NFAT–IL-2 axis in orchestrating intestinal immune homeostasis has been largely studied in terms of T helper-cell and Treg-cell biology^6^. The current literature supports that IL-2 is pivotal for maintaining tolerance to self and microbial antigens^8,9,18,22^, which is achieved via various mechanisms. First, CD4^+^ naïve T cells constitutively produce IL-2 in the intestinal mucosa, which induces and maintains the intestinal Treg-cell pool, as these cells are uniquely dependent upon IL-2^29^. Second, IL-2 can directly suppress the differentiation of pathogenic Th17 cells^30^. Although T-cell-derived IL-2 likely has an important role in promoting intestinal tolerance, our data also highlight the requirement for DC-derived IL-2 in this process. Of note, in the absence of ex vivo restimulation, the detected IL-2 signals in intestinal DCs are relatively weak. However, it is important to bear in mind that levels of IL-2 and intensity of the signal cannot be compared to the IL-2 levels in T cells. Like DCs, unstimulated T cells do have weak IL-2 transcription/production levels, which is dramatically boosted upon stimulation. In this scenario, low level IL-2 production by DCs seems to have an important biological function and can be directly used by naïve CD4^+^ T cells to differentiate into Treg cells thereby suppressing the differentiation of pathogenic Th17 cells. It is also possible that some regulatory feedback mechanisms might be in place in DCs to limit and fine tune the level of IL-2 expression, as very high levels could disrupt optimal function of these cells, as well as affecting their interaction with other cells such as CD4^+^ T cells. Using various in vivo models, we have shown that DC-derived IL-2 in the gastrointestinal tract plays a crucial biological role in maintaining the immune homeostasis. Thus, we are confident that the immune phenotype observed in our DC-IL-2-deficient mouse model was mostly due to reduction of IL-2 production by DCs.

We further analyzed the distinct contributions of innate and adaptive IL-2 to intestinal immune homeostasis. Here, we compared the systemic and intestinal phenotype of *Il2*^*CD11c*^ mice (lacking *Il2* in the myeloid compartment), *Il2*^*CD4*^ mice (lacking *Il2* in the CD4^+^ T cells), and *Il2*^*KO*^ mice (in which both myeloid and lymphoid *Il2* is absent). *Il2*^*CD4*^ mice exhibited IL-2 Deficiency Syndrome^24–26^ characterized by severe anemia, splenomegaly, uncontrolled expansion of T cells associated with a reduction of Treg cells, and loss of B cells. The same phenotypes noted in the *Il2*^*KO*^ model. Mice deficient in myeloid IL-2 (*Il2*^*CD11c*^ mice) did not develop the same pathology, but instead showed a transmural infiltration of mononuclear cells, without affecting CD8^+^ T cells compartment involved in erosive mucosal lesion observed in *Il2*^*KO*^ mice, indicating that lymphoid IL-2 is crucial for immune homeostasis in the peripheral immune tissues. The difference between *Il2*^*CD4*^ and *Il2*^*CD11c*^ is even more striking in the intestine, where loss of myeloid IL-2 resulted in a higher exacerbation of spontaneous colitis and a higher increase in the total number of leukocytes and activated CD4^+^ T effector cells producing IFNγ and IL-17 compared to mice lacking T-cell-produced IL-2. A significant reduction in the frequencies of Treg cells in the LP-colon of both *Il2*^*CD11c*^ and *Il2*^*CD4*^ mice was observed, indicating that both myeloid and adaptive IL-2 are equally important for the induction and/or maintenance of Treg cells in the mouse intestine^20,31^. Of note, the aberrant Treg:T effector-cell ratio that accompanied pathologic inflammation in *Cnb1*^*CD11c*^ and *Il2*^*CD11c*^ mice seems to be restricted to the intestine of these mice, as we did not detect any changes in Treg-cell frequency in the lung or axillary and popliteal lymph nodes, suggesting a fundamental role for the gut microbiota in the activation of this pathway and thus IL-2 production by intestinal myeloid cells.

The results presented here pose interesting questions for future research. It would now be interesting to investigate how DC-derived IL-2 mechanistically regulates the induction of intestinal Treg cells, as well as how it modulates effector T-cell functions in the mouse intestine. Moreover, the requirement of DC-derived IL-2 for the development and maintenance of innate cells, such as innate lymphoid cells, should be examined. Considering the differential effects of calcineurin signaling in various myeloid cell subsets, it will be important to investigate the effects of calcineurin inhibitors on different cell subsets in vivo. Calcineurin inhibitors are already in clinical use and elicit some benefits in patients with selected forms of IBD, including acute severe ulcerative colitis and steroid-refractory IBD. Data remain insufficient to recommend the use of calcineurin inhibitors (cyclosporin A or FK506) in Crohn’s disease^32^.

In summary, our data support that the calcineurin–NFAT–IL-2 pathway in intestinal DCs has a central role in eliciting proper T-cell responses in the mouse intestine under both steady-state and inflammatory conditions, by supporting Treg-cell induction and by suppressing excessive expansion of Th1/Th17 cells (directly or indirectly). We propose that therapeutic strategies that selectively boost DC-derived IL-2 production may have a beneficial effect in patients with IBD.

## Methods

### Mice

C57BL/6-*Ppp3r1*^tm1Stl^/J (*Cnb1*^*fl*/*fl*^, Stock No. #6581), B6.Cg-Tg(*Itgax*-cre)1-1Reiz/J (*CD11c*-cre, Stock No. #8068), B6.129P2-*Lyz2*^tm1(cre)Ifo^/J (*LyzM*-cre, Stock No. #4781), Tg(*Cd4*-cre)1Cwi/BfluJ (Stock No. #017336), and B6.129P2-*Il2*^tm1Hor^/J (Stock No. #2252) mice were obtained from The Jackson Laboratory, USA. C57BL/6 and *Rag2*^*KO*^ mice were obtained from the Biological Resource Center, Agency for Science, Technology and Research (A^*^STAR), Singapore. *Il2*^*fl*/*fl*^ mice were generated by Ozgene Pte^5^. All mice were maintained at the Biological Resource Center (A*STAR), under specific pathogen-free conditions. *Cnb1*^*fl*/*fl*^ mice were crossed with B6.Cg-Tg(*Itgax*-cre)1-1Reiz/J (CD11c-cre) and B6.129P2-*Lyz2*^tm1(cre)Ifo^/J (LysM-cre) mice. Mice lacking *Il2* specifically in CD11c^high^MHCII^+^ myeloid cells or CD4^+^ T cells were obtained by crossing *Il2*^*fl*/*fl*^ mice with B6.Cg-Tg(*Itgax*-cre)1-1Reiz/J (*CD11c*-cre) or Tg(*Cd4*-cre)1Cwi/BfluJ (CD4-cre) mice, respectively. *Il2* deletion was quantified in splenic CD11c^high^MHCII^+^ DCs and CD4^+^ T cells of *Cnb1*^*CD11c*^ mice, as previously described^5^. *Il2* deletion in *Rag2*^*KO*^*Il2*^*CD11c*^ mice was evident in the majority of CD11c^high^MHCII^+^ cells, but not in the CD11c^low^ compartment. Male and female mice, and congenic gender-matched littermate controls were used in all experiments.

All the experiments described in this study were allowed and carried out in accordance with the guidelines of the Institutional Animal Care and Use Committee of the Biological Resource Centre (A*STAR, Singapore). B6.TgIL-2-GFP mice^20^ were maintained in the animal facility at the Institut Pasteur (Paris, France) in accordance with European animal welfare regulations. These animal studies were approved by the Institut Pasteur Safety Committee and by the Paris Ethics Committee 1.

### Mouse models of colitis

Mice were exposed to 150 µl TNBS (Sigma-Aldrich) pre-sensitization solution (1% TNBS dissolved in 4:1 acetone:olive oil solution) that was applied to a shaved area of abdominal skin. Control mice were subjected to the same protocol with pre-sensitization solution lacking TNBS. After 7 days, mice were anesthetized by intraperitoneal ketamine/xylazine injection (100 µl/10 g body weight of 100 mg/ml ketamine and 20 mg/ml xylazine in saline) and then administered 1 mg TNBS (dissolved in 50% ethanol) intra-rectally using a 3.5 French catheter equipped with a 1 ml syringe. To ensure even distribution of TNBS throughout the entire colon and cecum, mice were held in an inverted vertical position for 30 s after instillation. In some experiments to induce colitis, naproxen (40 mg/kg/day) was administrated for 4 weeks via drinking water. The mice were then monitored daily for weight loss and fecal score. The criteria and numerical scores for macroscopic damage were as follows: 0, no ulcer, no inflammation; 1, no ulcer, local hyperemia; 2, colon wall thickening/edema; 3, ulceration and inflammation at one site only; 4, two or more sites of ulceration and inflammation. Additional points were scored for the presence of indurations, intestinal adhesions, and each additional site of inflammation or ulceration >1 cm^2^ in surface area.

### T-cell transfer model of chronic colitis

Splenocytes were collected from C57BL/6 mice aged 6–10 weeks, and naïve CD4^+^CD45RB^high^ T cells and CD4^+^CD45RB^low^CD25^+^ Treg cells were sorted by fluorescence-activated cell sorting (FACS). Naïve T cells (3×10^5^/mouse) were injected intravenously alone or in combination with Treg cells (1×10^5^/mouse) into *Rag2*^*KO*^*Il2*^*CD11c*^ and *Rag2*^*KO*^*Il2*^*fl*/*fl*^ recipient mice. Mouse weight was recorded to evaluate irritable bowel disease. Mice were then killed by CO_2_ asphyxiation at 110 or 140 days after adoptive cell transfer for macroscopic analysis, histopathological scoring and flow cytometric analyses of leukocytes.

### Intestinal permeability assay

Mice were fasted for 6 h and then administered 500 mg/kg FITC-dextran (4 kDa; Sigma-Aldrich) by oral gavage. Blood samples were collected 1 and 3 h later, centrifuged at 12,000 x* g* at 4 °C for 3 min, and the plasma was collected for analysis. The plasma was diluted in an equal volume of PBS and then analyzed for FITC-dextran concentration using a fluorescence spectrophotometer (485 nm excitation, 535 nm emission). A standard curve was obtained by diluting FITC-dextran in untreated plasma diluted with PBS (1:3 v/v).

### Inflammatory histological score

Sections of medial colon and small intestine were fixed in buffered formalin, cut (5 µm sections, ~150 µm between each section, 4–8 per fragment), and then stained with hematoxylin and eosin (H&E). Stained sections were examined and scored in a blinded fashion. The “degree of inflammation” was graded semi-quantitatively from 0 to 4: 0, no signs of inflammation; 1, very low level; 2, low level of leukocyte infiltration; 3, high level of leukocyte infiltration, high vascular density, and thickening of the colon wall; and 4, transmural infiltration, loss of goblet cells, high vascular density, and thickening of the colon wall. The “extent of inflammation” was graded from 0 to 4: 0, none; 1, mucosal; 2, submucosal; 3, mucosal and submucosal; 4, full thickness. Finally, the “involved surface area” was graded from 0 to 4: 0, none; 1, 0–25% of the mucosal surface; 2, 25–50%; 3, 50–75%; 4, 75–100%. The overall inflammatory score was obtained by summing the “degree” and “extent” scores, and multiplying by the ‘involved surface area’ (minimum score 0, maximum 32).

### Measurement of tissue inflammatory markers

Frozen fragments of small intestine and colon were homogenized using Tissue Protein Extraction Reagent (Thermo Scientific) and the homogenates were used to assess protein concentrations and cytokine levels. Neutrophil infiltration into the colon was monitored by measuring MPO activity using a spectrophotometric assay with tri-methylbenzidine (TMB) as the substrate and myeloperoxidase activity expressed as mU/mg of protein.

### IL-2-mAb complex treatment

IL-2-mAb complex was prepared by mixing 1 µg of recombinant IL-2 (Miltenyi Biotec) with 5 µg of anti-IL-2 mAb (clone JES6-1A12, eBioscience) in PBS per mouse for i.p. administration 3 per week, every 2 days for 4–5 weeks.

### DC transfer in vivo

Bone marrow cells from C57BL/6 CD45.1 or *Il2*^*KO*^ mice were cultured in conditioned media containing recombinant mouse granulocytes macrophages colony-stimulating factor (GM-CSF) or recombinant mouse Flt3-L for 11 days. CD103^+^ and CD11b^+^ DCs (CD11c^+^MHCII^high^) were FACS-sorted and adoptively transferred intravenously into 12-week-old *Cnb1*^*fl*/*fl*^ or *Cnb1*^*CD11c*^ mice at a ratio of 1:6 CD103:CD11b (total number of transferred cells: 5×10^5^). Two weeks post-transfer, mice were killed and the immune phenotype of mononuclear cells from spleen, SI, and colon was investigated by flow cytometry.

### Isolation of LP leukocytes from the colon

Isolated colons were cut longitudinally and divided into segments of 0.5–1 cm in length. The tissue segments pieces were then added to RPMI medium supplemented with 2% FBS, and incubated for 20 min at 37 ^o^C with constant stirring. After incubation, the resultant suspension was passed through a 70 µm sterile strainer and pieces of colon tissue were placed into a 50 ml tube containing 15 ml serum-free RPMI, shaken vigorously for 30 s, and then filtered twice through a 70 µm cell strainer. The remaining fragments of colon were washed in calcium-free and magnesium-free HBSS and treated with 1 mm EDTA in PBS (twice for 20 min) to remove the epithelium. The tissue was then digested with type-IV collagenase (0.8 mg/ml; Sigma-Aldrich) containing DNase and the leukocytes were enriched on a 40:75 Percoll gradient (Pharmacia) and the interface collected after centrifugation at 700 × *g* for 20 min.

### Isolation of LP leukocytes from the small intestine

Isolated SI was flushed with HBSS, the PP was removed, and then were cut longitudinally and divided into pieces of 1 cm in length. Epithelial cells were removed by incubation with 2 mm EDTA in HBSS supplemented with 10% FCS for 40 min at 37 °C, followed by vigorous shaking for 20 s. The samples were then collected and incubated with type-VIII collagenase (0.1 mg/ml, Sigma-Aldrich) and DNase I (50 µg/ml) in HBSS containing 10% FCS and 10 mm HEPES for 45 min at 37 °C. After digestion, the samples were shaken vigorously for 10 s, the supernatants were collected by filtration through a nylon mesh, and the tissue underwent a second round of enzymatic digestion. Leukocytes were enriched on a 40:75 Percoll gradient and the interface was collected after centrifugation at 700 × *g* for 20 min.

### Cell culture and stimulation

Splenic D1 DCs were cultured as described previously^33^. NFAT-luciferase reporter D1 cells (D1-NFAT-Luc) were generated using the Cignal Lenti NFAT Reporter (luc) kit (Qiagen) as previously described^5^. Briefly, D1 cells were grown to 80% confluence before transduction with Cignal Lenti NFAT reporter (MOI 5) using SureENTRY^TM^ Transduction reagent (2 μg/ml; Qiagen). Transduced D1 cells were selected in the presence of 150 μg/ml puromycin dihydrochloride (Sigma-Aldrich). Single D1 cell sub-cloning was carried out to generate a stable NFAT-luciferase reporter D1 cell line. D1-NFAT-Luc cells were seeded into a 96-well plate (5×10^4^ per well) and rested overnight prior to 30 min pre-treatment with cyclosporin A or tacrolimus (FK506) at the indicated concentrations. Cells were then stimulated for 4 h with CpG (10 μg/ml), polyI:C (10 μg/ml), LPS (10 ng/ml), soluble β-(1,3)-glucan (PGG; 1 μg/ml), whole glucan particles (WGP; 10 μg/ml) or thapsigargin (60 nm). NFAT nuclear translocation was detected using the ONE-Glo Luciferase Assay System (Promega) and the luminescence signal was quantified with the GloMax-Multi Detection System Luminometer module (Promega).

BM-derived DCs (5×10^5^ cells/ml) were cultured in Iscove’s modified Dulbecco’s medium containing GM-CSF (10 ng/ml), 10% heat-inactivated FCS, streptomycin (100 mg/ml) and penicillin (100 U/ml). The cells were harvested on day 6 and contaminating Gr-1^+^ cells were depleted using an AutoMACS apparatus (Miltenyi Biotec) prior to DC stimulation with LPS (10 μg/ml), zymosan (10 μg/ml) or thapsigargin (60 nm) for 18 h alone or in combination with pharmacological inhibitors of NFκB (BAY 11-7821, 1 µm) or p38 MAPK (SB 202190, 1 µm) added 30 min before stimulation.

Sorted CD11c^+^MHCII^high^ (CD11b^+^ and CD11b^−^) cells were seeded into 96-well plates (150,000 cells/100 µl/well) in complete RPMI medium supplemented with glutamine (2 mm), HEPES (10 mm) and β-mercaptoethanol (50 µm; all from GIBCO) and stimulated with thapsigargin (60 nm) for 16–20 h at 37 °C. Culture supernatants were then used for IL-2, IL-6, IL-10, IL-12p70, and IL-23p19 (eBioscience), and TGFβ1 (R&D System) ELISA.

### OTII cell conversion

Naïve congenic OTII cells (CD45.1^+^CD4^+^CD45RB^high^CD62L^+^CD44^low^) were sorted from spleens of donor mice and then transferred by intravenous injection into gender and age-matched *Rag2*^*KO*^*Il2*^*CD11c*^, *Rag2*^*KO*^*Il2*^*fl*/*fl*^, *Cnb1*^*CD11c*^, and *Cnb1*^*fl*/*fl*^ mice (1×10^6^ cells/mouse). After 24 h, mice were administered OVA protein (5 mg, Hyglos GmbH) dissolved in PBS daily for 7 days by oral gavage. The MLN were harvested on day 7 and OTII cells were analyzed for FoxP3, IFNγ, and IL-17 expression by flow-cytometry.

### Flow cytometry and sorting of cell populations

Cell suspensions prepared from spleen, LP, and MLN were subjected to flow cytometry or cell sorting. The anti-mouse antibodies were used at 1:100 dilution unless specified otherwise. CD3ε (clone 500A2), CD16/CD32 (clone 2.4G2), CD62L (clone MEL-14) were obtained from BD Pharmingen; CD4 (clone RM4–5) and CD11b (clone M1/70) were from eBioscience; CD11c (clone N418), CD44 (clone IM7), CD45 (clone 30-F11 used at 1:200 dilution), CD45R B220 (clone RA3-6B2), CD45RB (clone C36-16A), CD69 (clone H1.2F3), CD80 (clone 16-10A1), CD86 (clone GL1), CD103 (clone 2E7), I-A/I-E (MHCII, clone M5/114.15.2) were obtained from Biolegend. For Treg-cell quantification, cells were permeabilized using the Mouse FoxP3 Buffer Set (BD Bioscience) reagents, and labeled with a FoxP3 antibody (clone MF23). For NFAT-1 flow analysis staining, cells were fixed and permeabilized with BD Cytofix/Cytoperm™ Fixation/Permeabilization Solution Kit (BD Bioscience) and labeled with an anti-mouse-NFAT-1 (1:100, Rabbit anti-mouse IgG) for 1 h followed by labeling with an anti-rabbit IgG-FITC secondary antibody (1:500). For CD4^+^ T-cell intracellular cytokine labeling, cells were stimulated with PMA (1 µg/ml) and ionomycin (1 µg/ml) for 1.5 h before addition of Brefeldin A (10 µg/ml) and incubation for a further 5 h. The cells were then harvested and surface-labeled before permeabilization using BD Cytofix/Cytoperm™ reagents (BD Bioscience) and intracellular staining for 30 min at 4 °C with a 50 μl mix of anti-IL17 (clone TCII-18H101 used at 1:50 dilution) and anti-IFNγ (clone XMG1.2 used at 1:50 dilution). For cytokine release quantification, CD4^+^ T cells were sorted and then seeded into 96-well plates for stimulation with plate-bound anti-CD3 (5 µg/ml) and soluble anti-CD28 (2 µg/ml) in complete RPMI medium for 36 h at 37 °C. After incubation, the culture supernatant was analyzed for cytokine content by ELISA. For intracellular IL-2 labeling, DCs were incubated for 30 min at 4 °C with anti-CD45, anti-CD3, anti-CD11c, anti-MHC-II, and anti-CD103 antibodies. For intracellular labeling, cells were permeabilized using the BD Cytofix/Cytoperm™ Fixation/Permeabilization Solution Kit (BD Bioscience) and then incubated with anti-mouse-IL-2 antibody (clone JES6-1A12; eBioscience) (1:16, 20 µl/tube for 30 min at 4 °C) or the same amount of fluorescently conjugated Rat-IgG2a, as an isotype control.

### Immunofluorescent NFAT-1 staining

Cells were fixed in 2% paraformaldehyde for 10 min, washed with PBS and permeabilized with PBS containing 0.1% saponin, 0.2% gelatin, and 5 mg/ml BSA. The cells were then incubated with antibodies diluted in PBS containing 0.01% saponin and 0.2% gelatin. The following antibodies were used at a dilution of 1:500: NFAT-1 (cat. 4389S from Cell Signaling Technology) and CD11c PE (clone N418 from BioLegend). Cells were cytospun and mounted with Vectashield (Vector Laboratories). Images were acquired using a camera mounted on an Olympus FV1000 confocal microscope.

### Quantitative real‐time PCR

Total cellular RNA was extracted using the Arcturus^®^ PicoPure^®^ RNA Isolation Kit. High‐capacity cDNA Reverse Transcription Kits with RNase Inhibitor (Applied Biosystems) or the Super‐Script III First Strand Synthesis System for RT‐PCR (Invitrogen) were used for reverse transcription. Real‐time PCR was carried out with the following validated SYBR Green primer pairs: *Cnb1* forward 5′-TGTTCCGTGCCTTGAGGTTG-3′, reverse 5′-TCTGTTCCTTATCGCCTTTGAC-3′; *Nfat1* forward 5′-CTGGTCTACGGGGGCCAGCA-3′, reverse 5′-GGCAGGGACTGG GTGGTAGG-3′; *Nfat2* forward 5′-TGCAAGCCAAATTCCCTGGTGG-3′, reverse 5′-GGGGTCGGGAGGCATGGTGA-3′; *Il2* forward 5′-CCCAGGATGCTCACCTTC-3′, reverse 5′-CAACAGTTACTCTGATATTGCTGATG-3′; *Gapdh* forward 5′-TCGTCCCGTAGACAAAATGG-3′, reverse 5′-TTGAGGTCAATGAAGGGGTC-3′. Amplification was performed on a 7500 Real-Time QPCR system (Applied Biosystems) and the relative gene expression was calculated using the comparative Ct method (2^−ΔΔCt^).

### Statistical analyses

Data are expressed as the means ± standard error. One-way ANOVA was used for comparisons of more than two groups, followed by Tukey’s test for the analysis of naproxen-induced colitis, or by Dunnett’s multiple comparisons test to assess the levels of *Cnb1*, *Nfat1*, and *Nfat2* expression in CD11c-expressing cells compared with controls. A two-tailed, unpaired Student’s *t* test was used to compare two groups of data. *P* values < 0.05 were considered statistically significant. GraphPad Prism version 6 (GraphPad Software) was used to prepare graphics and perform the statistical analyses.

### Data availability

The authors declare that all data supporting the findings of this study are available within the article and its supplementary information files or from the corresponding authors on reasonable request.

## Electronic supplementary material

Supplementary Information(DOCX 11714 kb)
