# Supplementary material for: Calcineurin-mediated IL-2 production by CD11chighMHCII+ myeloid cells is crucial for intestinal immune homeostasis
Source: Nat Commun. 2018 Mar 16;9:1102. doi: 10.1038/s41467-018-03495-3 (PMC5856784; doi:10.1038/s41467-018-03495-3)
Supplement: Supplementary file 1 — Supplementary Information(DOCX 11714 kb) [file 41467_2018_3495_MOESM1_ESM.docx]

**Calcineurin-mediated IL-2 production by CD11c^high^MHCII^+^ myeloid cells is crucial for intestinal immune homeostasis**

Mencarelli et al.

**SUPPLEMENTARY FIGURES**

**
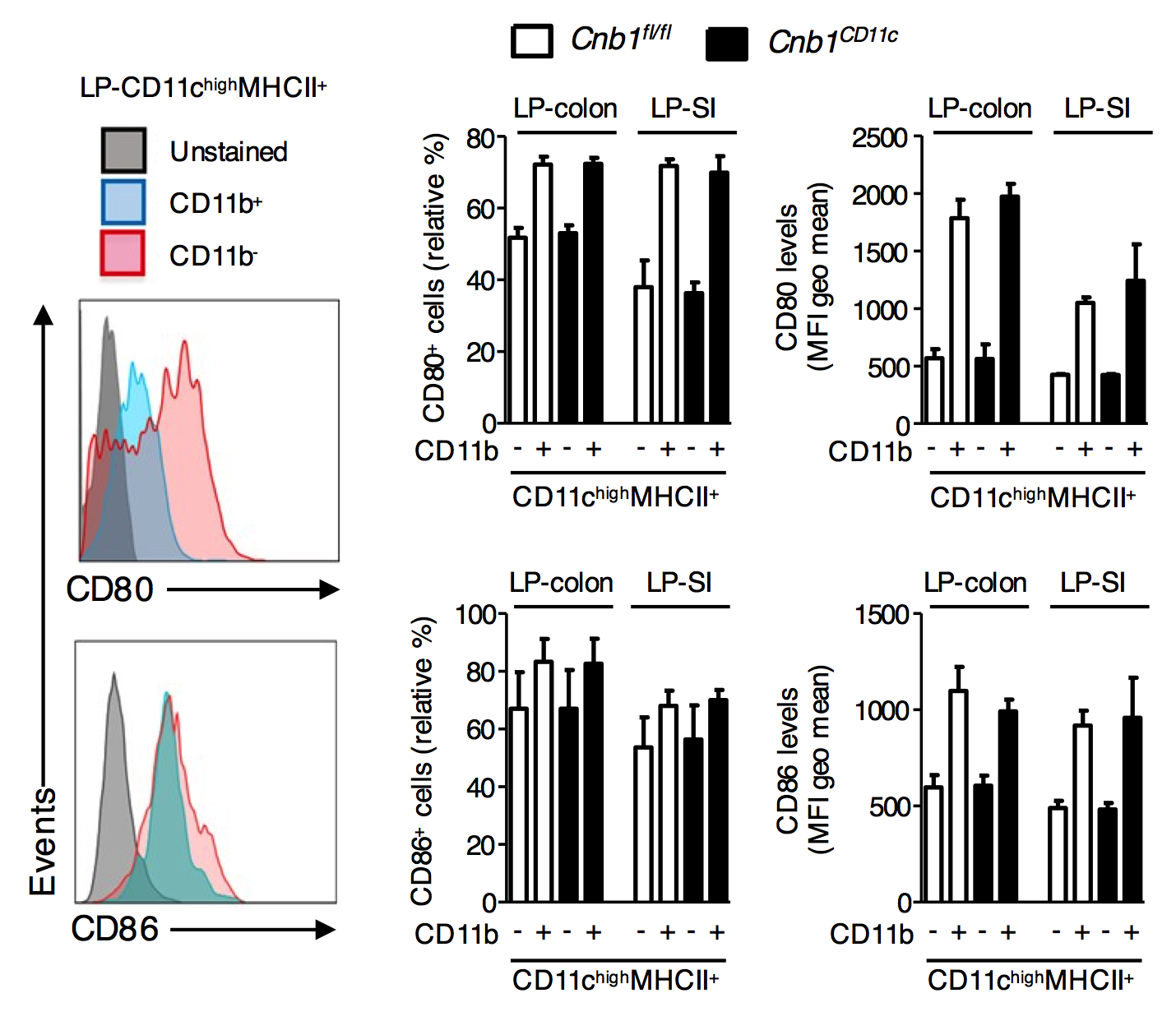
**

**Supplementary Fig. 1. Phenotypic analysis of cell-surface maturation markers on CD11c^high^MHCII^+^ (CD11b^+^ or CD11b^-^) cells.** Percentage and mean fluorescence intensity (MFI) of CD80 and CD86 expression in CD11c^high^MHCII^+^ populations (CD11b^+^ or CD11b^-^) in lamina propria (LP)-colon and LP-small intestine (SI) of *Cnb1^fl/fl^* and *Cnb1^CD11c^* mice. Representative histograms of CD80 and CD86 expression are shown (left). Data represent the means ± standard error of 3-4 experiments.

**
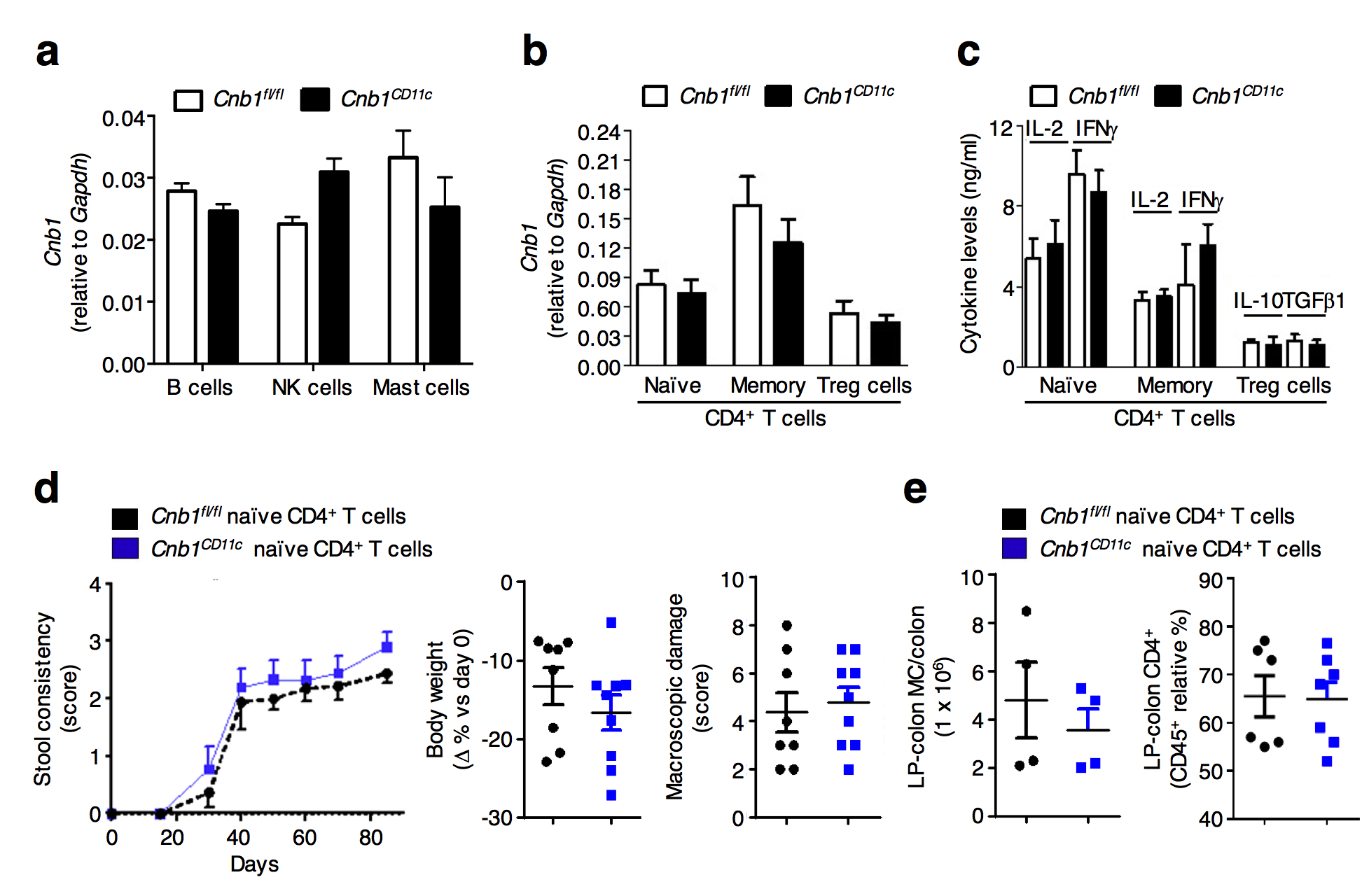
**

**Supplementary Fig. 2. Functional characterization of CD4^+^ T cells from *Cnb1^CD11c^* mice.** (**a**) Assessment of *Cnb1* expression by qRT-PCR in B, NK, mast cells, (**b**) naïve (CD45RB^high^), memory (CD45RB^low^CD25^-^) CD4^+^ T cells, and Treg cells (CD45RB^low^CD25^high^) sorted from MLN of *Cnb1^fl/fl^* and *Cnb1^CD11c^* mice. (**c**) Cytokine release from naïve, memory CD4^+^ T cells, and Treg cells stimulated with anti-CD3/CD28 antibodies was assessed by ELISA. Data represent the means ± standard error of three experiments (n = 5-7 mice/group per experiment, aged 6-10 weeks). (**d,e**) Colitis was induced in immunocompromised *Rag2^KO^* mice by adoptive transfer of naïve CD45RB^high^CD25^-^ CD4^+^ T cells isolated from the spleens of *Cnb1^CD11c^* and *Cnb1^fl/fl^* mice. Stool consistency, loss of body weight, macroscopic colon score (**d**), total leukocyte number and percentage of CD4^+^ T cells (**e**) in the LP-colon was used to assess the severity of inflammation. Data represent the means ± standard error of two experiments (n = 4-5 mice/group per experiment). Abbreviations: LP, lamina propria; MC, mononuclear cells.


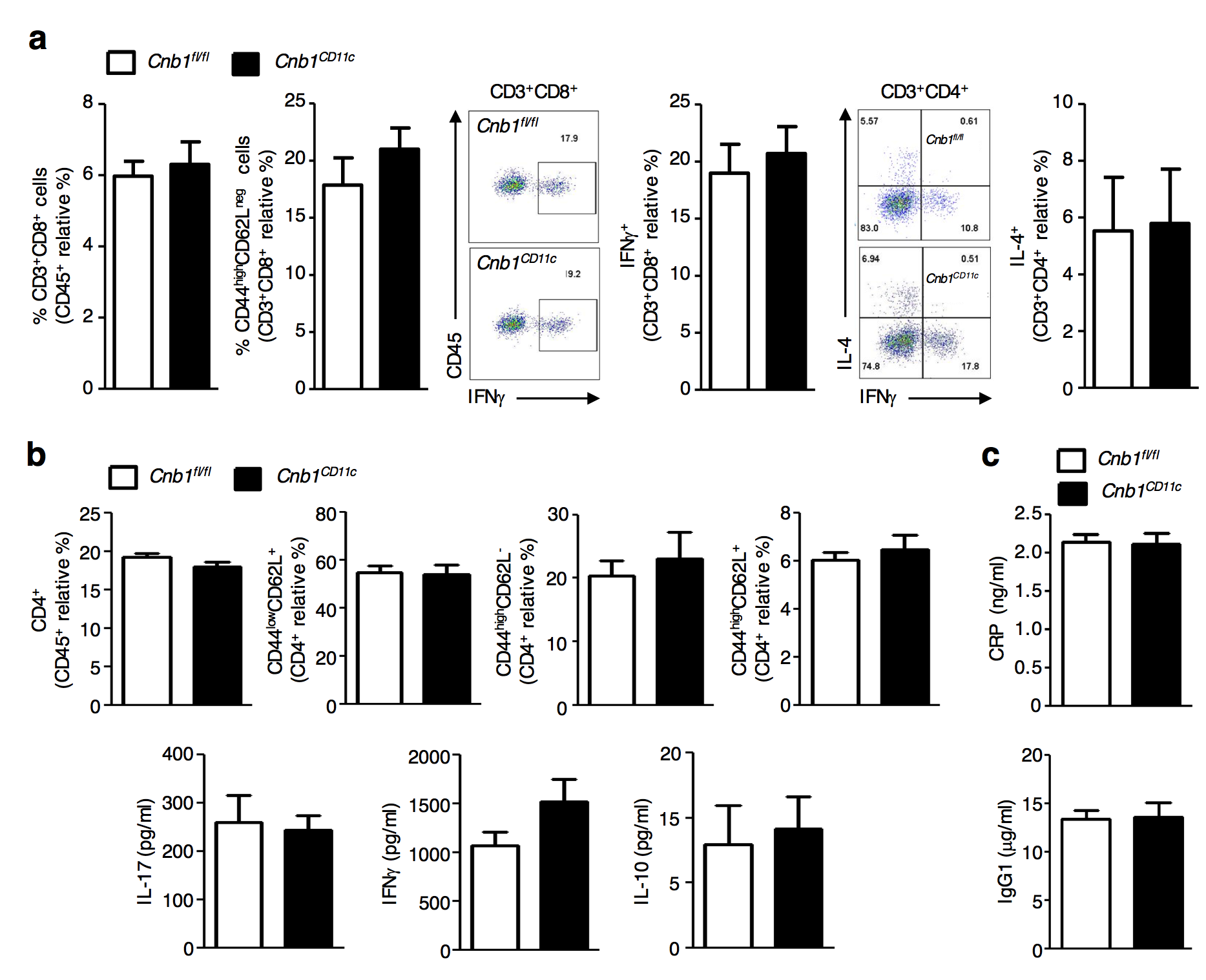


**Supplementary Fig. 3. Intestinal and systemic inflammatory markers in *Cnb1^fl/fl^* and *Cnb1^CD11c^* mice.** (**a**) Percentage of total CD8^+^ T cells relative to CD45^+^ leukocytes, and antigen-experienced CD44^high^ and IFNγ-producing CD8^+^ T cells from the lamina propria (LP)-colon of *Cnb1^CD11c^* and *Cnb1^fl/fl^* mice. Representative dot plots showing the percentage of IFNγ^+^ CD8^+^ T cells and IL-4^+^ CD4^+^ T cells in the LP-colon of individual *Cnb1^CD11c^* and *Cnb1^fl/fl^* mice are shown. Data represent the means ± standard error of two experiments (n = 5-7 mice/group per experiment). (**b**) Frequency of splenic naïve and antigen-experienced CD44^high^ CD4^+^ T cells, and cytokine production by splenocytes following 18 h *ex vivo* re-stimulation with anti-CD3/anti-CD28 monoclonal antibodies. (**c**) Markers of systemic inflammation (C reactive protein (CRP) and IgG1 titers in *Cnb1^CD11c^* and *Cnb1^fl/fl^* mice.

**
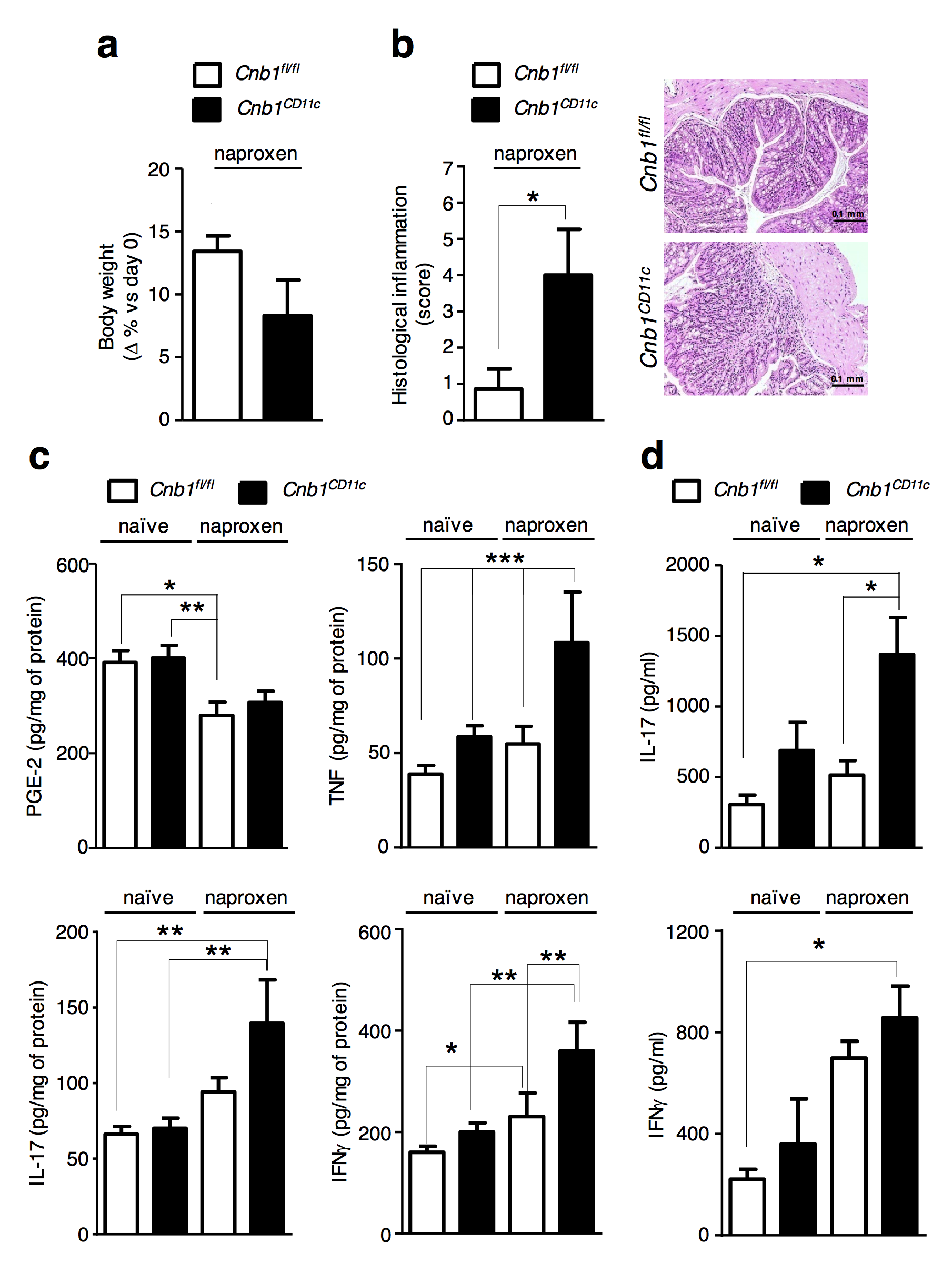
**

**Supplementary Fig. 4. Naproxen-induced colitis in *Cnb1^CD11c^* and *Cnb1^fl/fl^* mice.** (**a**) Body weight after 40 d naproxen treatment (expressed as % increase relative to baseline). (**b**) Histological analysis of colons of *Cnb1^CD11c^* and *Cnb1^fl/fl^* mice. Representative H&E-stained colon sections (10x magnification, scale bar 0.1 mm) illustrating the infiltrating leukocytes are shown. (**c**) Inflammatory markers in colon homogenates from *Cnb1^CD11c^* and *Cnb1^fl/fl^* mice. (**d**) IL-17 and IFNγ production by colonic lamina propria mononuclear cells from *Cnb1^CD11c^* and *Cnb1^fl/fl^* mice treated or not with naproxen, and re-stimulated for 36 h *ex vivo* with anti-CD3/anti-CD28 monoclonal antibodies. Data represent the means ± standard error of three experiments (n = 2-3 mice/group per experiment). *P <0.05, **P <0.01, ***P <0.001.

**
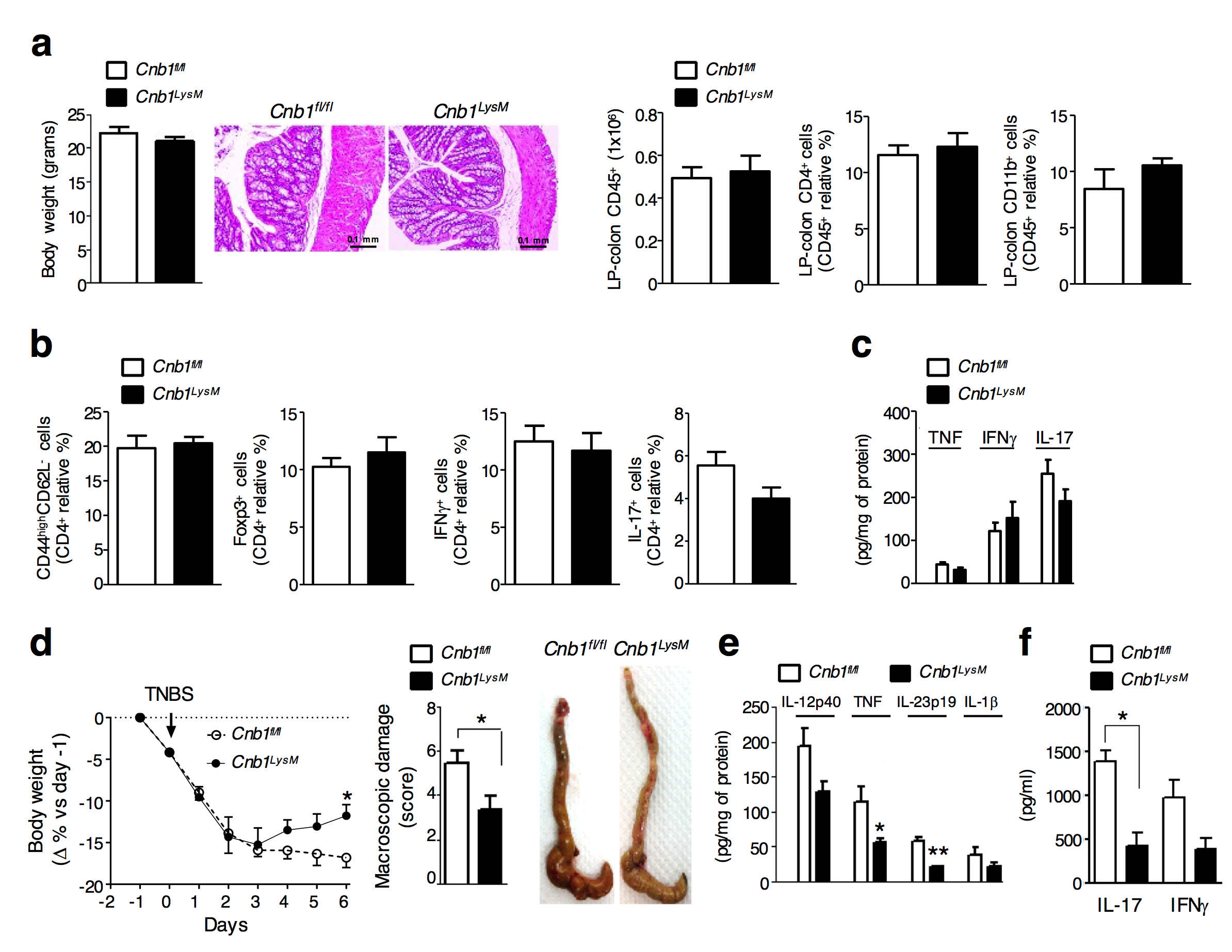
**

**Supplementary Fig. 5. Intestinal phenotype of *Cnb1^LysM^* mice under steady-state conditions and upon TNBS-induced colitis.** Body weight, histological analysis of the colon (10x magnification, scale bar 0.1 mm), percentage of total leukocytes, CD4^+^ T cells, CD11b^+^ myeloid cells (**a**), proportion of antigen experienced (CD44^high^CD62L^-^) CD4^+^ T cells, FoxP3^+^ Treg, IFNγ-producing and IL-17-producing CD4^+^ T cells (**b**) in the LP-colon of *Cnb1^LysM^* and *Cnb1^fl/fl^* mice. (**c**) Colonic mucosal levels of TNF, IFNγ and IL-17. Data represent the means ± standard error of two experiments (n = 4 mice/group per experiment, aged 10-12 weeks). (**d-f**) TNBS-induced colitis in *Cnb1^LysM^* and *Cnb1^fl/fl^* mice. Body weight, macroscopic analysis of the colon (**d**), mucosal cytokine levels (**e**) and IL-17 and IFNγ production by sorted CD4^+^ T cells from the mesenteric lymph nodes of TNBS-treated *Cnb1^LysM^* and *Cnb1^fl/fl^* mice re-stimulated *ex vivo* for 18 h with anti-CD3/CD28 monoclonal antibodies (**f**). Data represent the means ± standard error of two experiments (n = 6-7 mice/group per experiment, aged 8-12 weeks). *P <0.05, **P <0.01. Abbreviations: LP, lamina propria; TNBS, 2,4,6-trinitrobenzenesulfonic acid.

**
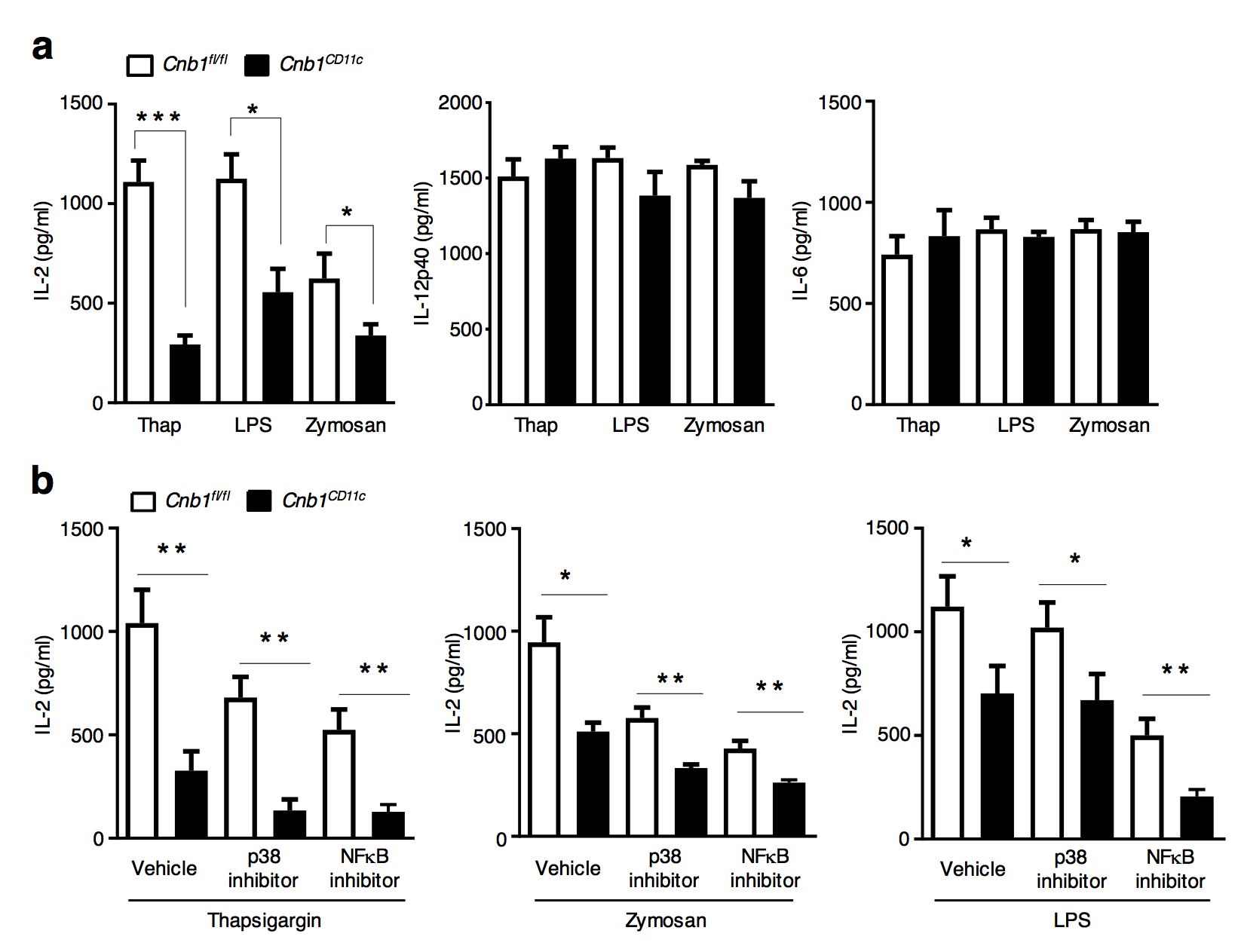
**

**Supplementary Fig. 6. Calcineurin B regulates IL-2 production from bone-marrow (BM)-derived dendritic cells (DCs) and is dependent on NFAT, NFκB and p38 MAPK signaling.** (**a**) BM-derived DCs were stimulated with thapsigargin (Thap), lipopolysaccharide (LPS) or zymosan for 16 h and IL-2, IL-12p40 and IL-6 were measured in culture supernatants by ELISA. (**b**) IL-2 release from BM-derived DCs pre-incubated for 30 min with NFκB or p38 MAPK inhibitors prior to stimulation with thapsigargin, LPS or zymosan for 16 h, as assessed by ELISA. Data are the means ± standard error of a representative experiment (n = 4) performed in triplicate. *P <0.05, **P <0.01, ***P <0.001.

**
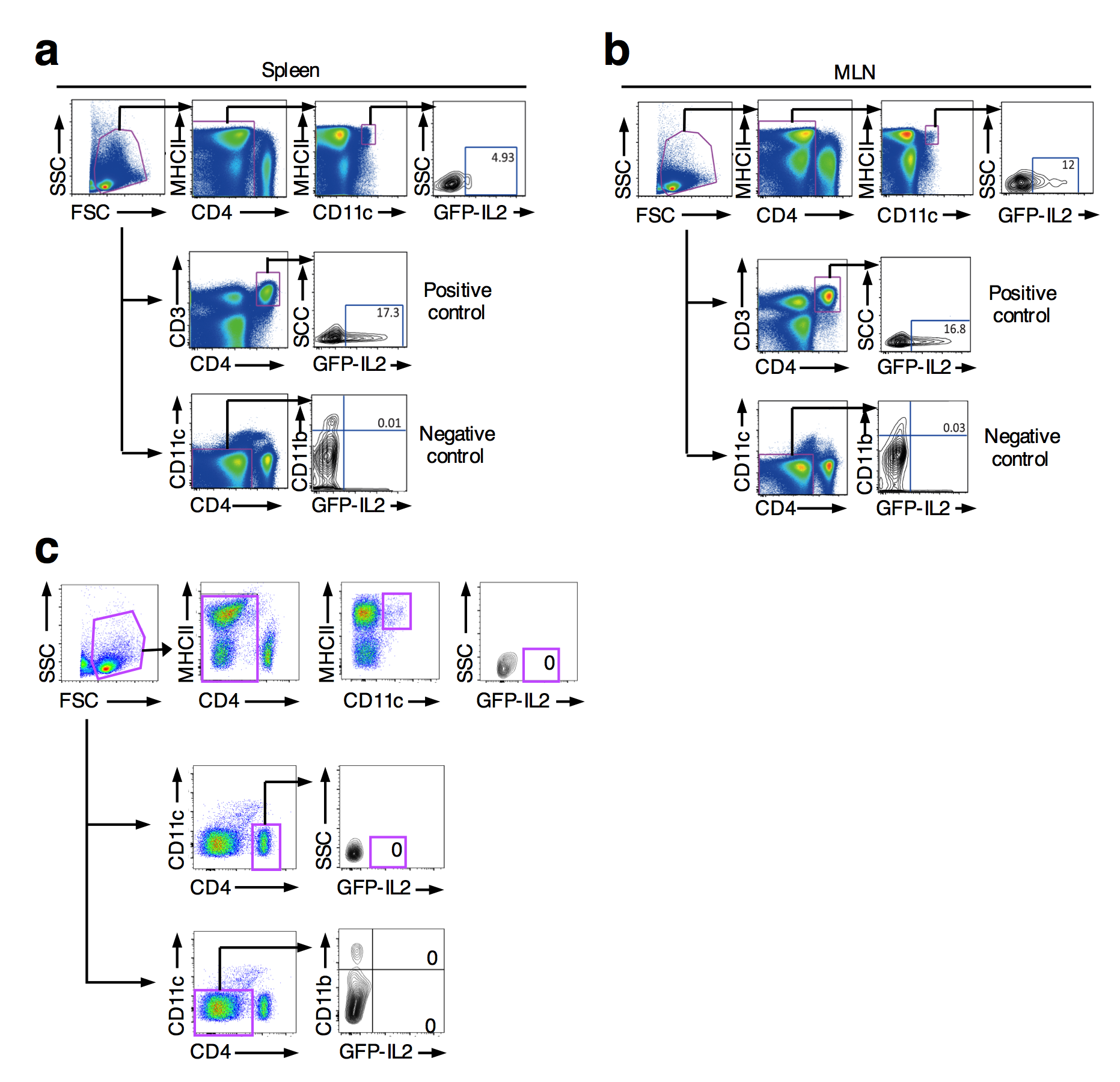
**

**Supplementary Fig. 7. IL-2 expression in CD11c^high^MHCII^+^ myeloid cells in spleen and mesenteric lymph node (MLN) of IL-2-GFP mice.** (**a,b**) Flow cytometric analysis of GFP
(IL-2) expression in CD11c^high^MHCII^+^ cells isolated from spleen (**a**) and MLN (**b**) of IL-2-GFP reporter mice. CD3^+^CD4^+^ T cells and CD4^-^CD11c^+^CD11b^+^ cells were used as positive (pos. ctrl) and negative (neg. ctrl) controls, respectively. (**c**) Splenic cells from C57BL/6 mice were used to determine the gating strategy for GFP^+^ cells.


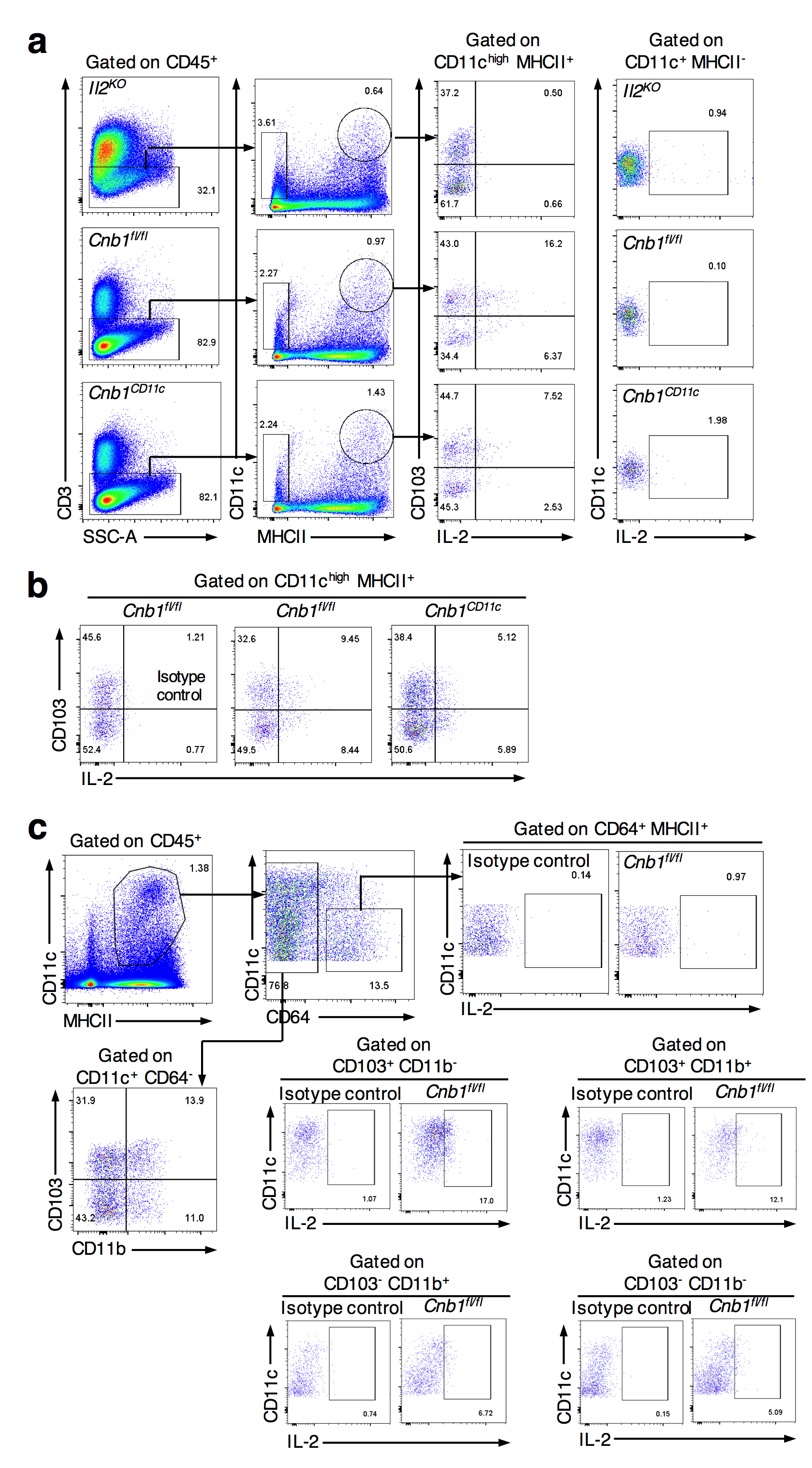


**Supplementary Fig. 8. IL-2 expression in CD11c^high^MHCII^+^ myeloid cells in the LP-colon of *Cnb1^fl/fl^* and *Cnb1^CD11c^* mice.** (**a**) Representative flow cytometry plots showing intracellular IL-2 staining of LP-colon CD45^+^CD3^-^CD11c^high^MHCII^+^ and CD45^+^CD3^-^CD11c^+^MHCII^-^ cells. The relative percentage of IL-2^+^ cells in the CD103^+^ and CD103^+^ subsets in *Cnb1^CD11c^*, *Cnb1^fl/fl^* mice (aged 6-10 weeks) and *Il2^KO^* mice (aged 4 weeks) is shown. (**b**) IL-2 intracellular staining of total colonic CD45^+^CD3^-^ CD11c^high^MHCII^+^ cells plotted versus CD103 expression from *Cnb1^fl/fl^* and *Cnb1^CD11c^* mice compared to the same population stained with an IL-2 isotype antibody. (**c**) Representative images of IL-2 intracellular staining in DCs and CD64^+^ macrophages isolated from LP-SI of *Cnb1^fl/fl^* and *Cnb1^CD11c^* mice compared with the same population stained with an IL-2 isotype antibody. Abbreviations: ab, antibody; LP, lamina propria.


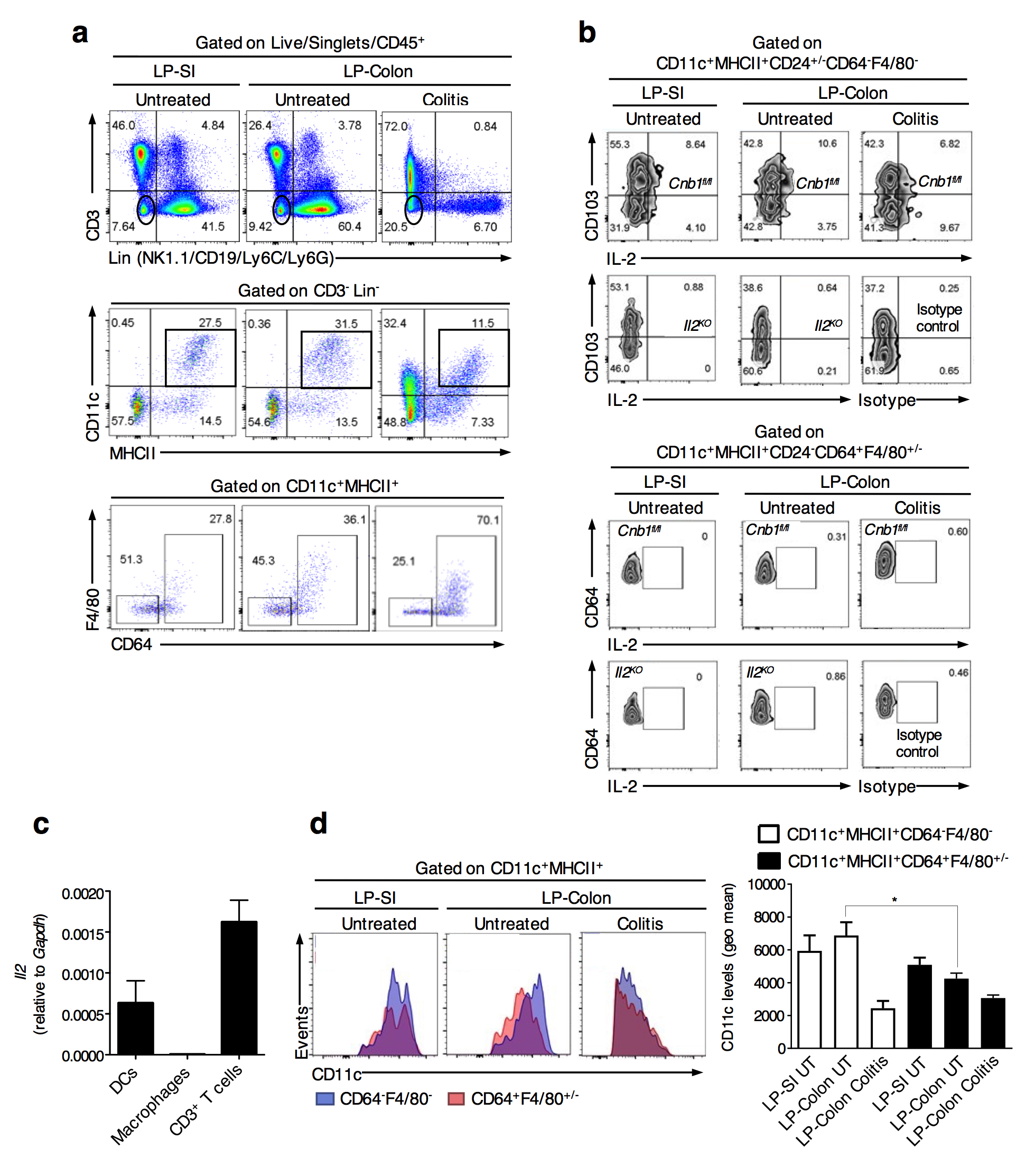


**Supplementary Fig. 9. IL-2 expression in intestinal macrophages and DCs during steady-state and colitis conditions.** (**a**) Gating strategy used to identify *bona fide* macrophages (CD45^+^Lin^-^CD3^-^CD11c^+^MHCII^+^CD64^+^F4/80^+/-^) and DCs (CD45^+^Lin^-^CD3^-^CD11c^+^MHCII^+^CD24^+/-^CD64^-^F4/80^-^) isolated from LP-colon and LP-SI of *Cnb1^fl/fl^* mice (aged 6-10 weeks) at steady-state, or during colitis induced in immunocompromised *Rag1^KO^* mice by transfer of naïve CD4^+^ T cells (CD45RB^high^CD62L^+^CD44^-^CD25^-^). (**b**) Representative flow cytometry plots showing intracellular IL-2 labelling of LP-intestine DCs and macrophages plotted versus CD103 expression. Dot plots of LP-intestine DCs and macrophages obtained from *Il2^KO^* mice (aged 4-5 weeks) and from the same populations stained with an IL-2 isotype antibody were included as controls. (**c**) Assessment of *Il2* expression by qRT-PCR in *bona fide* macrophages (CD45^+^Lin^-^CD3^-^CD11c^+^MHCII^+^CD64^+^F4/80^+^), DCs (CD45^+^Lin^-^CD3^-^CD11c^+^MHCII^+^CD64^-^F4/80^-^) and CD3^+^ T cells (CD45^+^Lin^-^MHCII^-^CD3^+^) sorted from LP-colon of C57BL/6 WT mice (aged 6-10 weeks, n = 2 experiments; 5 mice/exp) at steady-state. (**d**) Representative histograms showing CD11c expression on intestinal DCs and macrophages. Bar graph shows mean ± SEM of the indicated cell populations (pooled results of 3 experiments, n = 5-6/group).


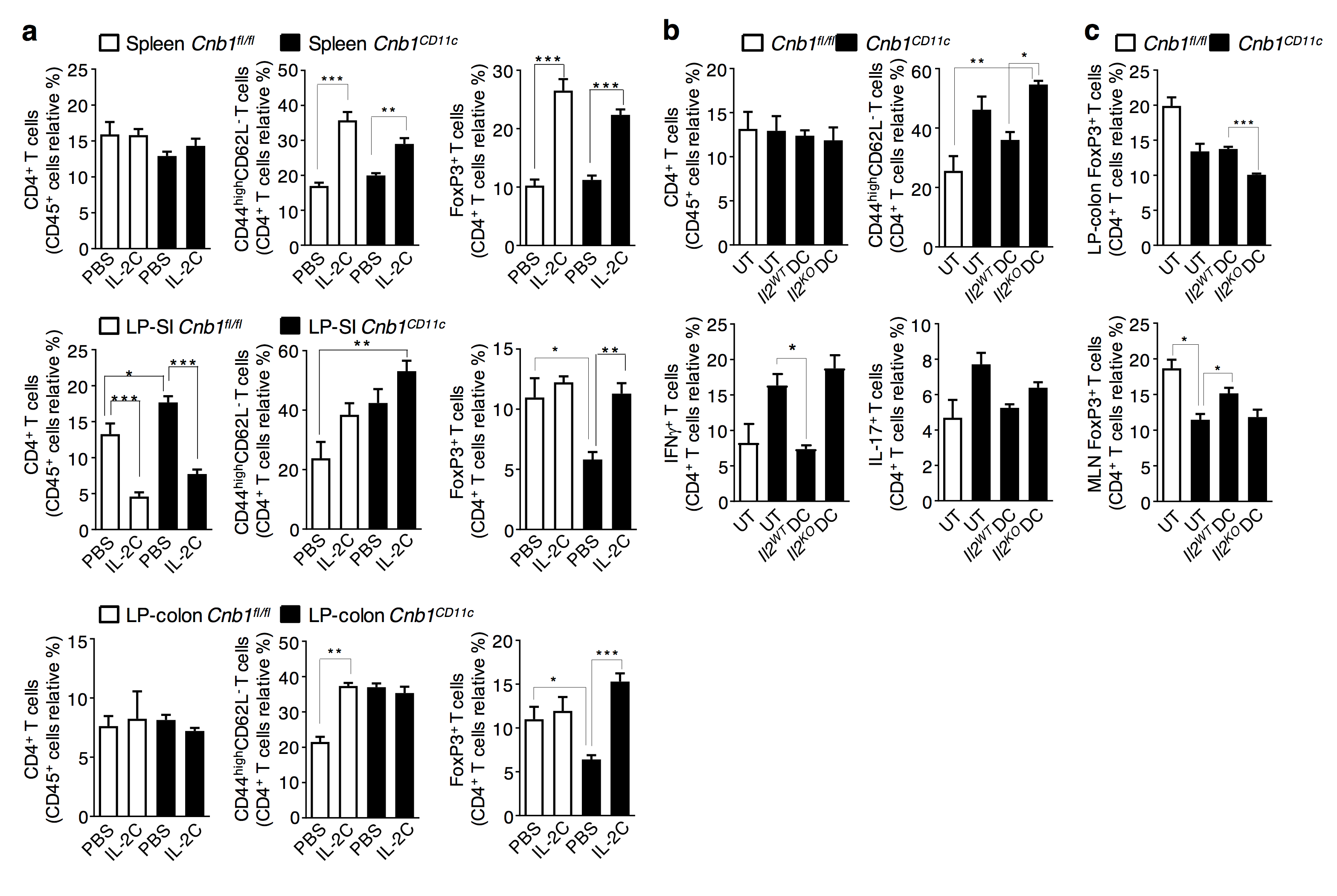


**Supplementary Fig. 10. Immune phenotype of CD4^+^ T cells in IL-2 reconstituted *Cnb1^CD11c^* mice.** (**a**) *Cnb1^CD11c^* and *Cnb1^fl/fl^* mice were treated with an IL-2 complex (IL-2C) and the phenotype of CD4^+^ T cells obtained from spleen, LP-SI and LP-colon of mice was assessed four weeks after treatment by flow cytometry analysis. Data are the means ± standard error of four mice per conditions. (**b,c**) *Cnb1^CD11c^* mice were adoptively transferred with *Il2^WT^* or *Il2^KO^* DCs. Percentage of total, antigen-experienced CD44^high^CD62L^-^, and IFNγ-producing or IL-17-producing CD4^+^ T cells obtained from LP-colon of *Cnb1^CD11c^* mice receiving intravenously *Il2^WT^* or *Il2^KO^* DCs for two weeks. Proportion of FoxP3^+^ Treg cells was evaluated in LP-colon (above) and MLN (below) of DC-transplanted *Cnb1^CD11c^* mice. Untreated *Cnb1^CD11c^* and *Cnb1^fl/fl^* mice were included as controls. Data represent the means ± standard error of three mice/group. *P <0.05, **P <0.01, ***P <0.001. Abbreviation: MLN, mesenteric lymph node; UT, untreated.

**
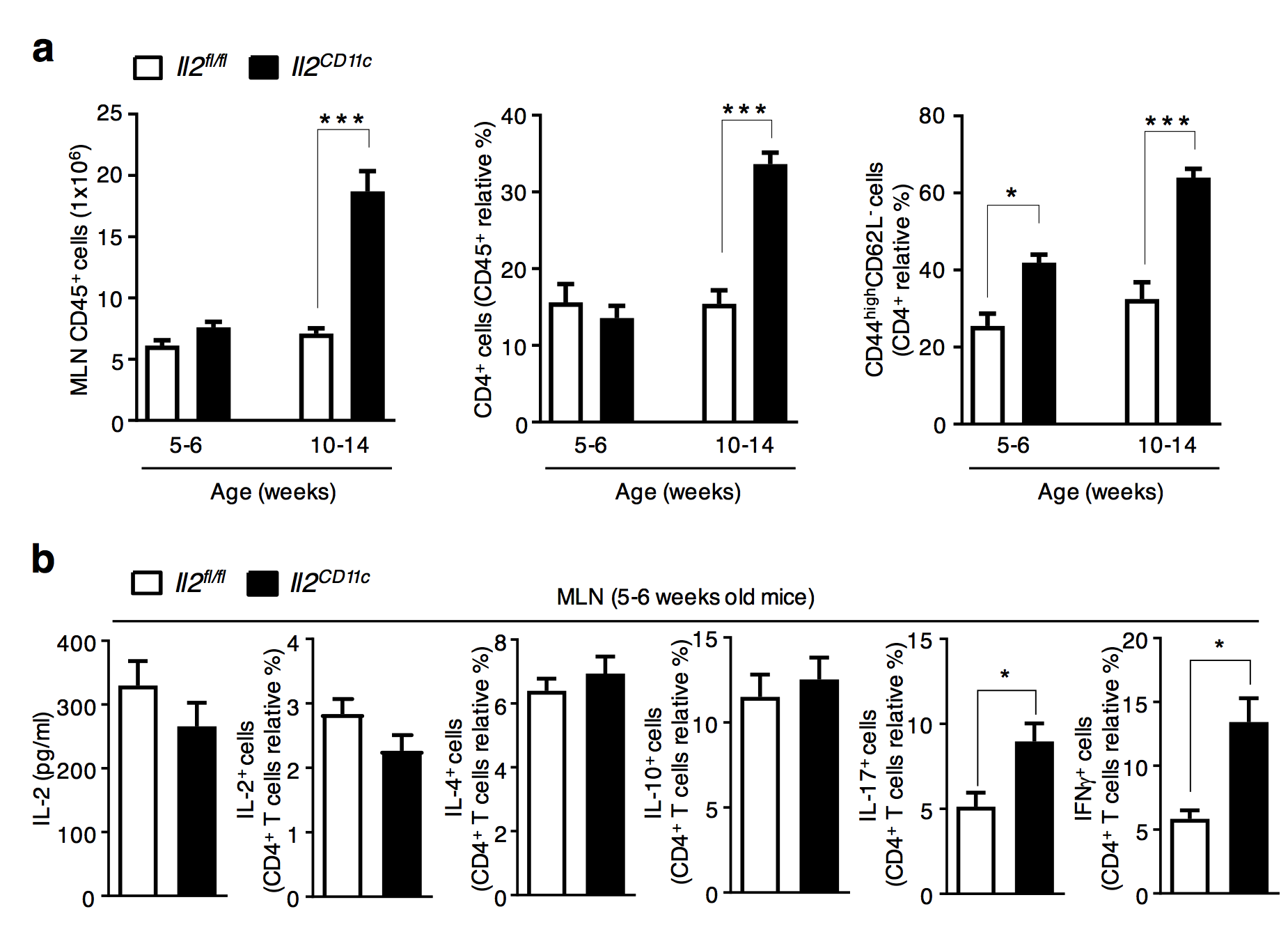
**

**Supplementary Fig. 11. Immune phenotype of MLN mononuclear cells from *Il2^CD11c^* and *Il2^fl/fl^* mice.** (**a**) Total number of mononuclear cells obtained from the MLN of *Il2^CD11c^* and *Il2^fl/fl^* mice at 5-6 and 10-14-weeks-old. The percentage of total and antigen-experienced CD44^hi^CD62L^-^ CD4^+^ T cells is shown. (**b**) MLN CD4^+^ T cells of *Il2^CD11c^* and *Il2^fl/fl^* mice were re-stimulated *ex vivo* for 18 h with anti-CD3/CD28 mononuclear antibodies and cytokine levels were measured by flow cytometry and ELISA (only for IL-2). Data represent the means ± standard error of 3-4 experiments (n = 2-3 mice/group per experiment). **P* <0.05, ****P* <0.001. Abbreviation: MLN, mesenteric lymph node.

**
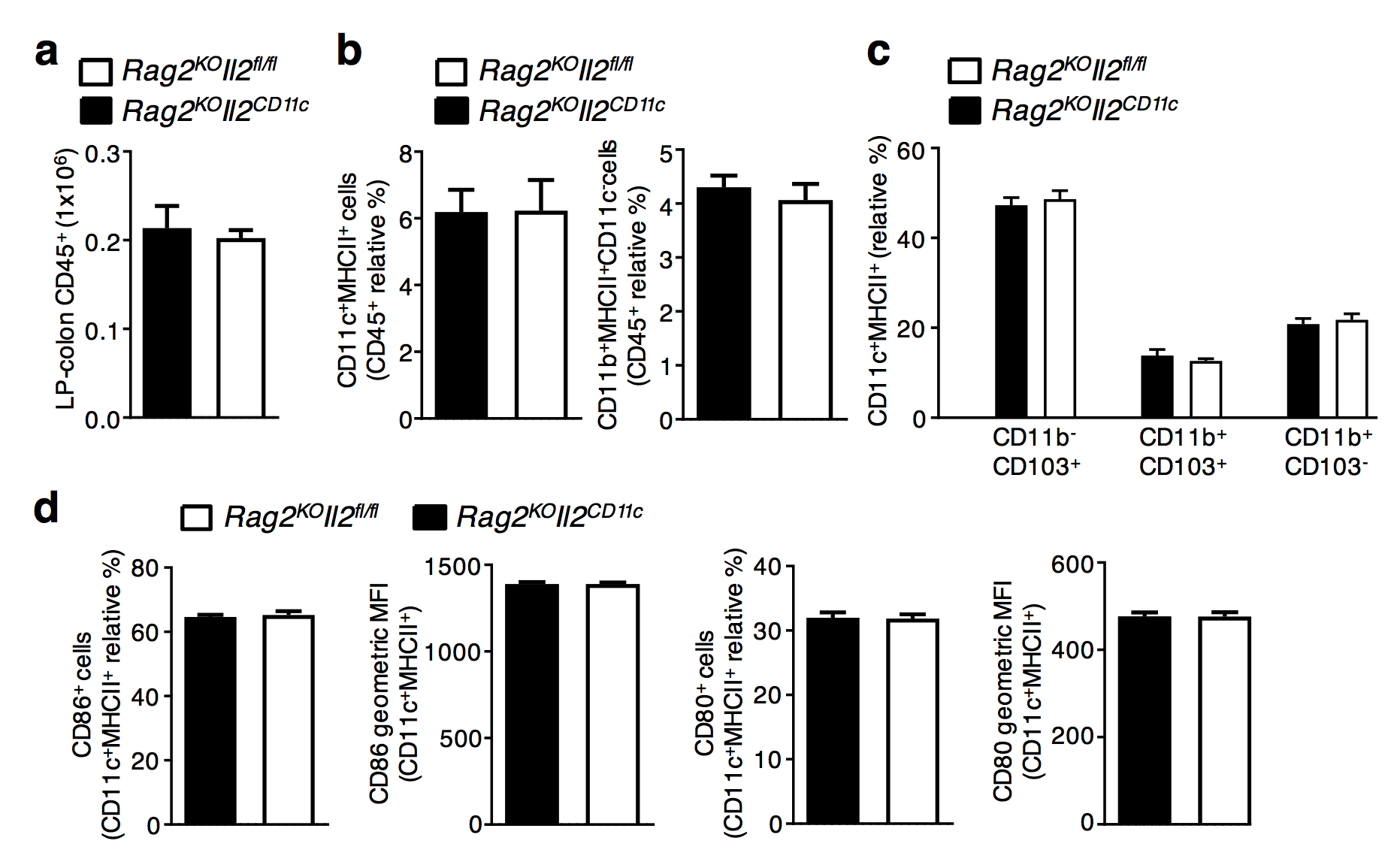
**

**Supplementary Fig. 12. *Il2* deletion in CD11c^high^MHCII^+^ cells does not affect the composition of myeloid cells in LP-colon of mice.** Frequency of total CD45^+^ leukocytes (**a**), CD11c^+^MHCII^+^ (including the three subsets CD11b^-^CD103^+^, CD11b^+^CD103^+^ and CD11b^+^CD103^-^) and CD11b^+^MHCII^+^ cells (**b,c**) obtained from LP-colon of *Rag2^KO^Il2^fl/fl^* and *Rag2^KO^Il2^CD11c^* mice. (**d**) CD80 and CD86 expression in CD11c^+^MHCII^+^ cells was evaluated as percentage and mean fluorescence intensity. Data represent the means ± standard error of two experiments (n = 2 mice/group per experiment, aged 10-13 weeks).

**
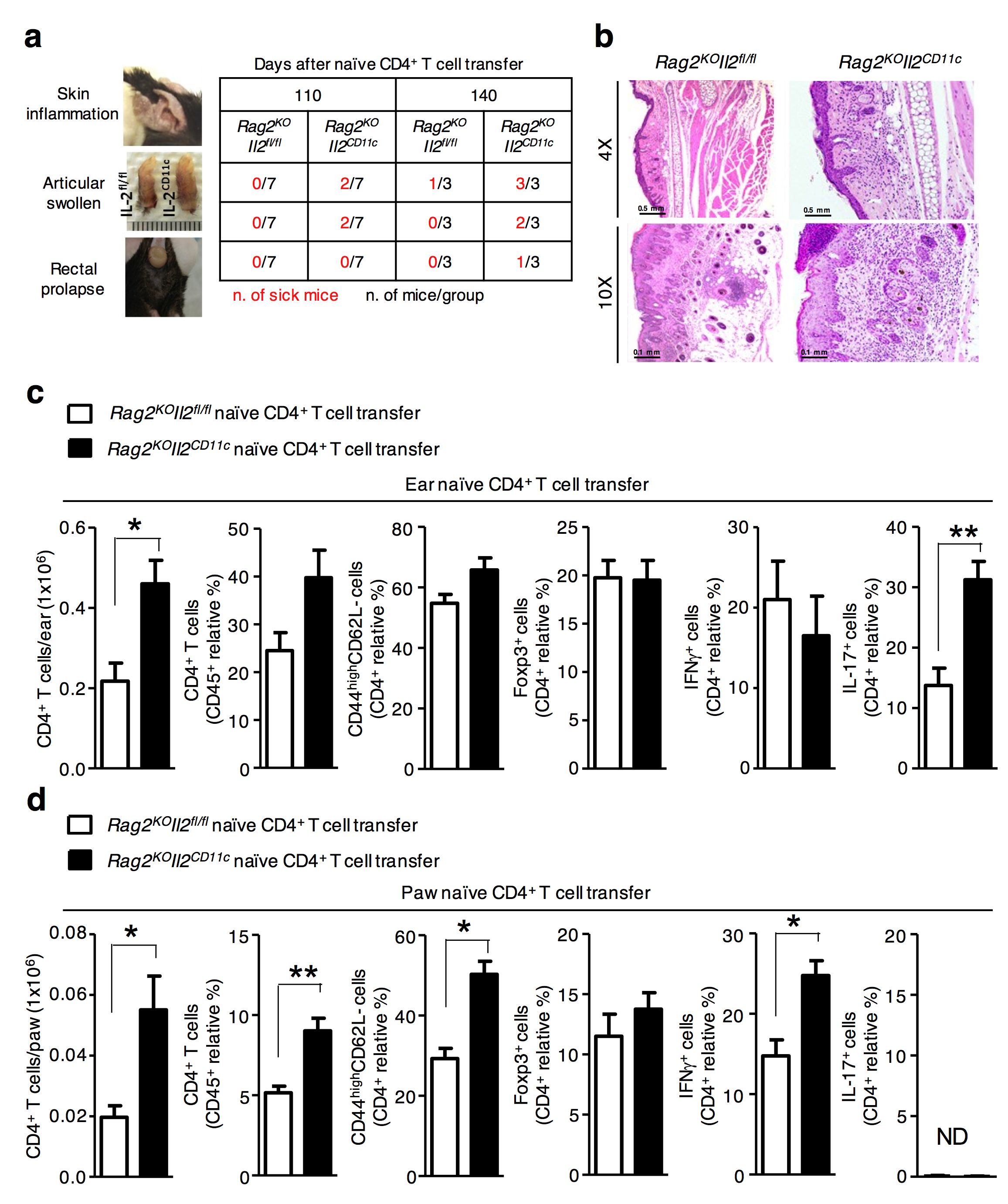
**

**Supplementary Fig. 13. Colitis-related extra-intestinal manifestations induced by adoptive transfer of naïve CD4^+^** **T cells into the *Rag2^KO^Il2^fl/fl^* and *Rag^KO^Il2^CD11c^* mice.** (**a**) Characterization of dermatological, articular and rectal phenotypes of *Rag2^KO^* mice with or without *Il2* deletion (*Rag2^KO^Il2^CD11c^* and *Rag2^KO^Il2^fl/fl^*, respectively) 110 and 140 days post-adoptive transfer of wild-type splenic naïve CD4^+^ T cells. The number of mice in which the extra-intestinal phenotypes were observed out of the number of total mice is indicated in the table. (**b**) Representative images of H&E-stained ears (magnification 4x, scale bar 0.5 mm and magnification 10x, scale bar 0.1 mm) showing macroscopic skin lesions in *Rag2^KO^Il2^CD11c^* and *Rag2^KO^Il2^fl/fl^ mice*, 90 days after induction of colitis. (**c, d**) Phenotype of immune cells isolated from the ear (**c**) and anterior paw skin (**d**) of *Rag2^KO^Il2^CD11c^* and *Rag2^KO^Il2^fl/fl^* mice, 90 days after T-cell transfer. The total number and the percentages of total, activated (CD44^high^CD62L^-^) CD4^+^ T cells and FoxP3^+^ Treg cells, as well as IL-17- or IFNγ-producing CD4^+^ T cells is shown. Data represent the means ± standard error of 2-3 experiments (n = 2-3 mice/group per experiment). **P* <0.05, ***P* <0.01.
